# Uncharted waters: the unintended impacts of residual chlorine on water quality and biofilms

**DOI:** 10.1038/s41522-020-00144-w

**Published:** 2020-09-25

**Authors:** Katherine E. Fish, Nik Reeves-McLaren, Stewart Husband, Joby Boxall

**Affiliations:** 1 Sheffield Water Centre, Department of Civil and Structural Engineering, University of Sheffield, Sheffield, S1 3JD UK; 2 NERC Biomolecular Analysis Facility, Department of Animal and Plant Sciences, University of Sheffield, Western Bank, Sheffield, S10 2TN UK; 3 Department of Materials Science and Engineering, University of Sheffield, Sheffield, S1 3JD UK

**Keywords:** Applied microbiology, Biofilms, Water microbiology, Microbial ecology, Microbiome

## Abstract

Disinfection residuals in drinking water protect water quality and public heath by limiting planktonic microbial regrowth during distribution. However, we do not consider the consequences and selective pressures of such residuals on the ubiquitous biofilms that persist on the vast internal surface area of drinking water distribution systems. Using a full scale experimental facility, integrated analyses were applied to determine the physical, chemical and biological impacts of different free chlorine regimes on biofilm characteristics (composition, structure and microbiome) and water quality. Unexpectedly, higher free chlorine concentrations resulted in greater water quality degredation, observable as elevated inorganic loading and greater discolouration (a major cause of water quality complaints and a mask for other failures). High-chlorine concentrations also reduced biofilm cell concentrations but selected for a distinct biofilm bacterial community and inorganic composition, presenting unique risks. The results challenge the assumption that a measurable free chlorine residual necessarily assures drinking water safety.

## Introduction

Anthropogenic attempts to manage our natural and built environments can create selective pressures that have unexpected consequences, potentially posing environmental or public health risks. For example, the introduction of cane toads to control sugar-cane beetles causing widespread ecological disruption^1^, or antimicrobial usage promoting “superbug” emergence^2,3^. Often the unintended consequences are driven by (micro)biological responses to ecological selection pressures. This is increasingly relevant when considering microbial management, with respect to disease control, microbial contamination and/or biofouling. Microorganisms are predominantly found in biofilms: multi-species communities adhered to surfaces via self-produced extracellular polymeric substance (EPS) matrices, into which (in)organics can be incorporated. The presence, characteristics and mobilisation of biofilms (and associated material) in any environment can pose aesthetic, biological and chemical risks. Hence, understanding and managing biofilms is a priority.

Safe drinking water is essential to protect public health. Treated water is typically distributed to consumers via heterogenic networks of pipes and ancillaries: the Drinking Water Distribution System (DWDS). During distribution, physical, chemical and microbiological water quality degrades, engendering water quality failures; an issue for consumers and water suppliers. Globally, drinking water discolouration (evidenced by elevated turbidity, of which iron is a predominant contributor) is a leading symptom of water quality failure, commonly causes customer complaints^4^ and can mask other failures, including microbial concerns. Discolouration is driven by material accumulated at the pipe wall being mobilised into the bulk water: analogous to biofilm accumulation and detachment^5^. Recurrence of discolouration highlights the need for risk mitigation, yet this can be costly and disruptive, including flushing strategies and mechanical cleaning to remove material^4^.

Disinfectant residuals are commonly maintained within DWDS ostensibly to limit microbial regrowth in the bulk water during transportation, thus protecting water quality (and public health). Internationally, free chlorine is the most commonly used residual, although some systems employ monochloramine, particularly where organic loads remain high monochloramines may limit disinfection by-product (DBP) formation^6^. There are, however, exceptions, such as the Netherlands, parts of Germany, Austria and Switzerland^7^, where no residual is used. This is commonly driven by disinfection benefits being outweighed by risks associated with potentially carcinogenic DBPs, which are of growing concern with increasing detection/identification^6,8^. An article by Speight et al^9^. calculated the contact times required to inactivate various microorganisms (assuming 0.5 mgL^−1^ chlorine, pH7 and 5 °C), concluding that the disinfectant contact times within DWDS are likely ineffective in their inactivation. These sub-lethal doses of disinfectant can then exert a selective pressure and various research studies have shown that the presence, type and concentration of disinfection impacts the planktonic bacterial composition of drinking water, enriching or decreasing certain functional genes or bacterial taxa^10–12^. Similarly, comparison of regulatory water sample compliance illustrated that the USA, which uses chlorine residual disinfection, had 10 times more total coliform failures than the Netherlands, which does not use a disinfection residual^9^, this is after adjusting for population, although there are other differences beside disinfection between the systems (e.g. infrastructure age, organic concentrations). However, biofilms account for the majority of microbial loading within DWDS, not planktonic cells, and monitoring bulk water alone will lead to constituents of the DWDS microbiome being overlooked^13^. A series of studies, comparing biofilms from a chloraminated DWDS (in the USA) with samples from a system where no residual is used (in Norway), demonstrated a difference between the planktonic and biofilm microbiomes within both systems, as well as difference in biofilm bacterial composition between the disinfected and non-disinfected systems^13–15^. Although there were differences other than disinfectant residual between the systems (notably water source and water treatment which are known to impact downstream microbiomes), the research demonstrated the potential for disinfectant residuals to impact biofilm ecology within operational DWDS^13–15^. Studies such as these highlight the need to control environmental variables in order to isolate and determine the impact of disinfection concentration on biofilms and water quality more clearly, as differences in pipe infrastructure and the water quality will influence the impact of disinfectant agents.

Whilst chlorine has been shown to generally attenuate bacterial biofilm cell concentrations^16–18^, it is important to note that less, or no, inhibitory effect of chlorine has been reported for other taxa such as eukaryotes, particularly fungi^16,19,20^. Additionally, biofilm-bound microorganisms have greater disinfection tolerance than their planktonic counterparts. The mechanisms behind this resistance are debated^21^, but EPS is recognised as integral to biofilm disinfection protection, as well as mechanical stability^21,22^ and can influence DBP formation^23^. It is crucial to recognise that EPS (and associated particles) are integral to biofilms and that a reduction in bacterial quantities with increasing chlorine concentration does not necessarily translate to decreases in these other biofilm components or a reduced likelihood of water quality degradation if biofilm is mobilised – this is an area which requires further investigation. Nevertheless, a common perception remains in the water industry and public domain that chlorine residuals will limit biofilm accumulation in its entirety, therefore reducing biofilm associated risks to water quality and public health (such as discolouration and any associated microbial mobilisation).

This study aimed to ascertain the impacts of residual chlorine concentrations on DWDS biofilm characteristics and discolouration response. Specifically, we aimed to establish if the action of free chlorine residuals to suppress planktonic regrowth applied to biofilms, and determine any subsequent (unintended) impacts on water quality. A key element of the work was to implement holistic analysis integrating physical, chemical and biological parameters to better understand chlorine, biofilm and water quality interactions within DWDS.

## Experimental design and overview

**Fig. 1:**
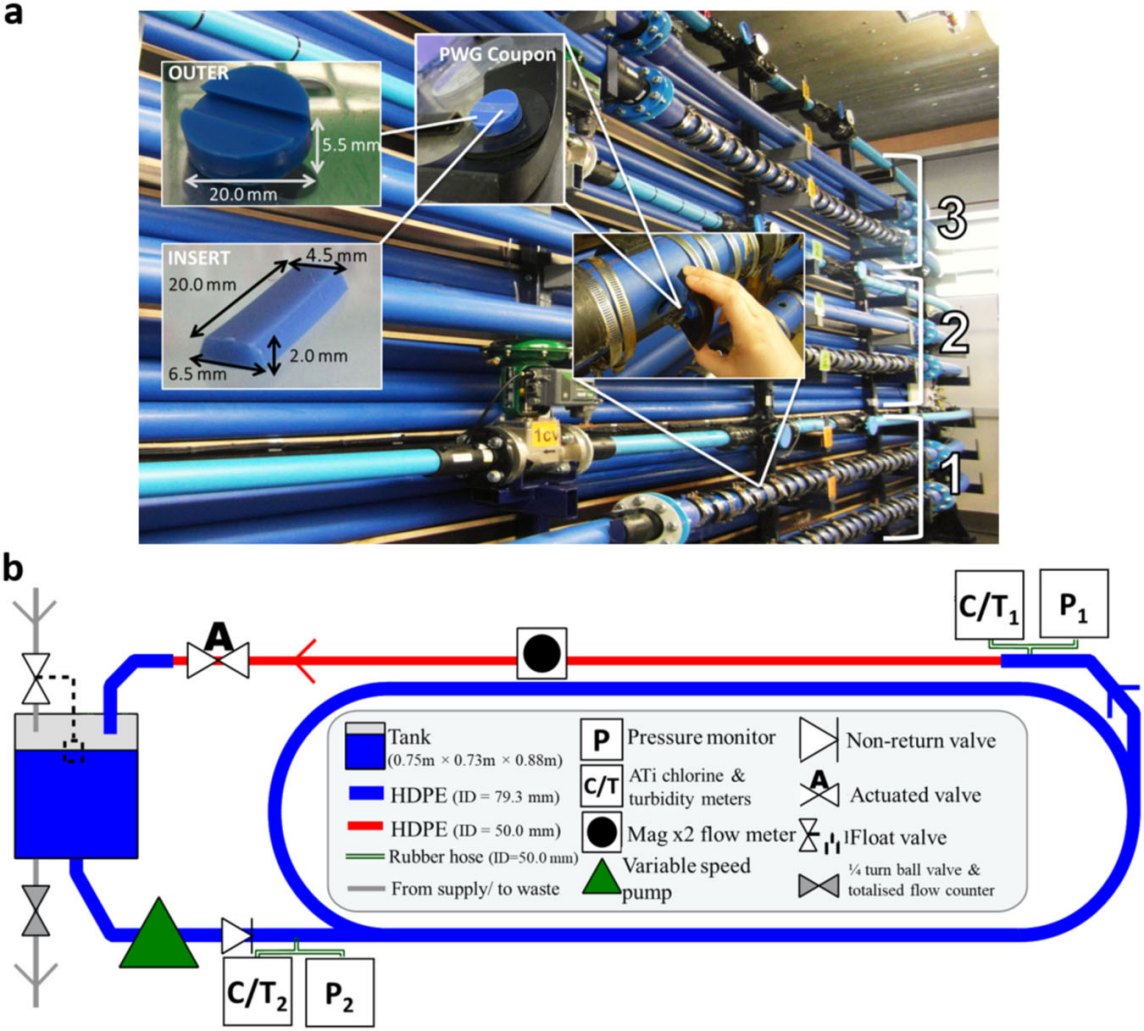
Drinking Water Distribution System (DWDS) experimental facility. **a** The three independent high density polyethylene loops, each 203 m long, ensuring pipe effects dominate, with Pennine Water Group coupons (comprising outers and inserts) facilitating biofilm sampling^25,35,36^. Water quality was monitored using online metres and spot samples. **b** Schematic of one loop showing: enclosed reservoir tank, pump, type of pipelines, valves and monitoring equipment. C/T_1_ denotes the location of turbidity and chlorine metres during growth, C/T_2_ indicates the relocation of a set of metres (from one of the other loops) during flushing to enable extra monitoring of the loop being flushed.

In order to generate rigorous data and hence robust knowledge relevant to operational DWDS, biofilms were developed within a laboratory based full scale DWDS experimental system (Fig. 1). This facility is internationally unique as it enables controlled conditions that are fully representative of operational drinking water networks (temperature, hydraulic regime, including diurnal flow patterns–Supplementary Fig. 1, pressures, materials–high density polyethylene, HDPE, is the most commonly used material for modern DWDS repair and implementation^24^, water chemistry, ecology, surface to volume ratio and exchange mechanisms) combined with online monitoring and PWG coupons^25^ for biofilm sampling (see Methods section: DWDS experimental facility). The system is supplied from the local network (high organics upland catchment, ferric based treatment and cast iron trunk mains) via connection directly to the trunk main. This facility uniquely enables laboratory level environmental manipulation, experimental replication and a robust sampling regime of water quality and biofilms while accurately simulating the environmental conditions of operational DWDS. Note that the experimental set up reported herein was the same as used in a previous study by the authors to investigate the impacts of chlorine on microbial community succession^16^. The present study addresses fundamentally different questions, provides extended biofilm characterisation beyond just the microbiome, but does draw on some data points from the author’s previous paper.

To address the aims of this study, biofilms were developed over a 28-day growth phase, under one of three chlorine regimes (Supplementary Fig. 2): High-chlorine (boosted to the top-end of U.K. limits for taste and odour; 0.80 mgL^−1^ ± 0.16), Medium-chlorine (control; 0.45 mgL^−1^ ± 0.05) or Low-chlorine (dechlorinated; 0.05 mgL^−1^ ± 0.06). Subsequently, the discolouration response of each regime was determined by applying an intervention, termed “flushing”, during which flow rates were increased incrementally (thus elevating shear stress) and water quality was monitored to detect any subsequent changes (see Methods section: Flushing and discolouration response). These two phases comprised Test 1. The test was then immediately repeated such that biofilms were regrown for an additional 28 days under the Low (0.03 mgL^−1^ ± 0.05), Medium (0.35 mgL^−1^ ± 0.05) and High (0.82 mgL^−1^ ± 0.05) chlorine regimes, after which the same flushing process was applied as was used in the first test. These repeated phases comprised Test 2. Water quality exhibited natural variation during the growth phases of both tests but all the parameters monitored were within U.K. standards (Supplementary Table 1). Only free- and total-chlorine concentrations consistently differed (*p* < 0.01; *χ*^2^ ≥ 34) between regimes (Supplementary Fig. 1 and Supplementary Table 1), leading to a slightly lower oxidation redox potential (ORP) in Low-chlorine at the end of the growth phase of Test 1 only (*p* = 0.01, *χ*^2^ = 10), ORP did not differ between regimes in Test 2. As expected, increasing free-chlorine concentration reduced bulk-water cell counts (Supplementary Table 1).

In both Test 1 and Test 2, biofilms were sampled (from each of the chlorine regimes) prior to flushing (i.e. at the end of the growth phase) and at the end of the flushing phase; these sample points will be referred to as Pre-Flush and Post-Flush, respectively. To indicate the test from which they were sampled the test number will be added, e.g. Pre-Flush1 to indicate Pre-Flush samples from test 1.

## Results and discussion

### Chlorine residual impacted discolouration

**Fig. 2:**
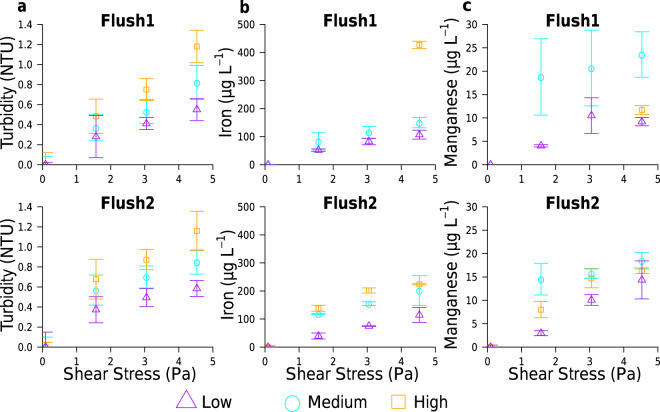
Discolouration responses to elevated shear stress during the flushing of the chlorine regimes. Discolouration was determined primarily by **a** Turbidity (506 ≤ *n* ≤ 1091) with consideration of **b** Iron (*n* = 3) and **c** Manganese (*n* = 3) concentrations. Flush1 refers to the flushing phase of test 1, Flush2 indicates data from the flushing phase of test 2. Data normalised to well-mixed concentrations (0.09 Pa) of each system, mean ± standard deviation plotted. Linear regressions in each plot had *R*^2^ values of **a** 0.82 ≤ *R*^2^ ≤ 0.99, **b** 0.89 ≤ *R*^2^ ≤ 1.00 and **c** 0.76 ≤ *R*^2^ ≤ 0.98. High-chlorine: metal concentrations only available for final flushing step for Flush1. Chlorine regimes differed in their turbidity (ANCOVA on raw data: *F* ≥ 2869, *p* < 0.001), iron (ANCOVA on raw data: *F* ≥ 26, *p* < 0.001) and manganese (ANCOVA on raw data: *F* ≥ 10, *p* ≤ 0.003) responses.

Unexpectedly, flushing of the High-chlorine system (by incrementally increasing the flow rate) produced a significantly greater discolouration response (assessed via turbidity) than the Medium- or Low-chlorine systems across all stages of flushing, of both tests (Fig. 2). Compared to the other regimes, the High-chlorine system also had a greater final concentration of iron (known to be associated with discolouration) at the end of Flush 1 and a greater rate of iron mobilisation during Flush 2 (Fig. 2). Conversely, the Low-chlorine regime consistently resulted in the lowest impact on water quality with the lowest discolouration and metal concentrations. Even after just 28 days of growth, material was mobilised from the High-chlorine regime at sufficient volumes to approach or breach the water quality standards for discolouration and iron concentrations (Fig. 2 and Supplementary Table 1). This contradicts the common perception of residual chlorine impacts on water quality and also studies of cast iron pipes, which suggest increasing oxidant concentration (disinfectant or dissolved oxygen) in drinking water decreases iron release^26,27^. Although surprising, High-chlorine repeatedly resulted in the greatest discolouration and Low-chlorine the least; as observed during the flushing of test 1, test 2 (Fig. 2) and preliminary tests (Supplementary Fig. 2).

Irrespective of chlorine regime, the bulk water turbidity, iron and manganese concentrations increased significantly during flushing, as shear stress increased (Fig. 2; other physio-chemical parameters did not differ significantly). Manganese (also associated with discolouration) was consistently mobilised at lower concentrations than iron, but, similarly to iron, greater manganese concentrations were mobilised from High- and Medium-chlorine than Low-chlorine biofilms (Fig. 2). However, manganese concentrations showed the least change between chlorine regimes, compared to turbidity or iron. Critically, the different discolouration responses were not driven by the rate of (in)organic supply because the chlorine regimes had equivalent hydraulics (governing rate of supply and transfer to biofilms) and bulk water quality (e.g. iron, manganese, TOC concentrations), indicating this trend is process driven/limited. These results could be considered to infer that chlorine use should be decreased or eliminated to reduce the possibility of a measurable discolouration response occurring. However, the application of machine learning to historical water quality data highlighted that destabilisation of scale can occur when oxidant concentrations are low^28^. Also, chlorine plays several critical roles in DWDS and water treatment, namely microbial inactivation and limiting planktonic regrowth. Therefore, it would be naïve to withdraw chlorine without better consideration of the drivers causing the discolouration differences described herein, which requires characterisation and understanding of the (in)organics (i.e. biofilm) at the pipe wall, including the impact that chlorine has upon them.

### Biofilm inorganics

**Fig. 3:**
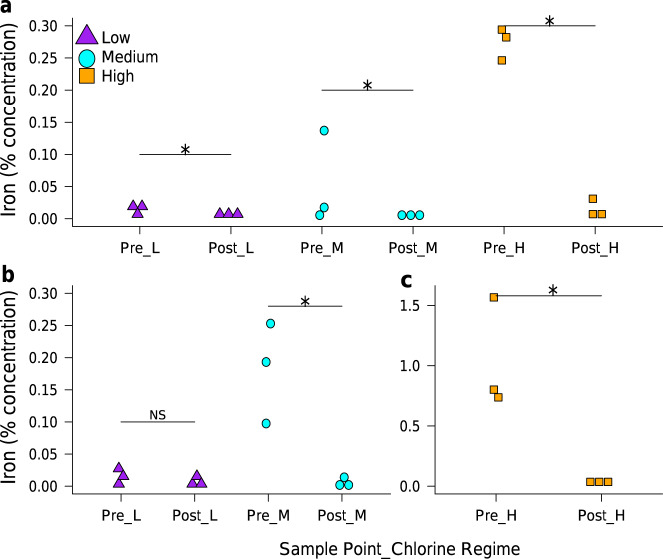
Quantification of iron in biofilms from the Pre- and Post-flush phases of the three chlorine regimes. **a** Flush1, **b**, **c** Flush2. L Low, M Medium, H High; Asterisk significance determined via Wilcox-1-tailed tests (*W* = 9, 0.03 ≤ *p* ≤ 0.05), NS = not significant (*W* = 6.5, *p* = 0.24). Chlorine regimes differed at Pre-flush1 and Pre-flush2 *χ*^2^ ≥ 5.65, *p* ≤ 0.05. *N.b*. different *y*-axis scale in **c**.

Greater accumulation (and subsequent mobilisation) of iron occurred in High-chlorine biofilms compared to Medium- or Low-chlorine biofilms, with the latter having the least (Fig. 3), mirroring the bulk water discolouration responses (Fig. 2). Various elements were detected at the pipe wall (Supplementary Fig. 3), analysis of total elemental fingerprints highlighted iron and chlorine as the main inorganic descriptors for differences between biofilms. Manganese was only detectable in three High-chlorine biofilms (Methods section: cell concentration analysis), where, similarly to the bulk water trends, concentrations were lower than iron. As the biofilms were formed within HDPE pipes no leaching of iron would occur from the pipe wall into the biofilm, rather the input of iron in to the system is as a trace inorganic within the incoming source water. This is a possible limitation of this study, in that cast iron pipes are known to promote different bacterial communities to plastic pipes^29^, so there may be a need to research the effects of chlorine residual specifically within cast iron pipes. However, the current study is useful in that it facilitates exploration of the specific role of biofilms (in isolation) in concentrating iron and acting as a sink/source of inorganics within DWDS.

Biofilms contained less iron after flushing than prior to flushing (irrespective of chlorine regime; Fig. 3 and Supplementary Fig. 3) providing direct evidence that the iron mobilised into the bulk water originated from the pipe wall. This release of entrapped metals from the pipe wall has been previously inferred based on increases in bulk water metal concentrations during the flushing of pipelines^4,5^ (a trend also observed in the current study) but not conclusively determined.

Metal concentrations in the bulk water were the same between chlorine regimes throughout the growth phases of both test 1 and test 2. Therefore, elevated iron and manganese concentrations at the pipe wall must have been driven by processes expedited in the High-chlorine regime. Chlorine is an oxidative agent, therefore higher residual chlorine may have promoted metal precipitation causing greater accumulation. However, the kinetics of free chlorine oxidising metals is generally slow in relation to the typical hydraulic retention times of water systems as described by Knocke et al^30^. High-chlorine did not have a significantly greater ORP than Medium-chlorine and only differed slightly from Low-chlorine during test 1, which seems to confirm that the differences in metal accumulation were not dominated by chemical oxidation in response to the different regimes. Oxidation of metals such as iron or manganese can occur due to microbial oxidation as well as chemical oxidation; a review of manganese oxidation specifically mentions the uptake of manganese by media support biofilms^31^, and metal oxidation is known to occur due to intracellular uptake by oxidising organisms which have been detected in DWDS^32^ or via adsorption to EPS molecules^33^. Additionally, iron concentrations were greater in Pre-Flush2 biofilms than Pre-Flush1 biofilms, (a trend most pronounced in High-chlorine), despite no significant differences in ORP between the growth phases of test 1 and test 2. This further refutes the oxidation/chemically driven precipitation theory and suggests that additional processes were governing the differential iron accumulation, such as microbially driven processes due to different EPS or microbiome compositions in test 2 compared to test 1. Furthermore, during flushing of test 1, Medium-chlorine had a greater discolouration response than Low-chlorine, yet these regimes had similar Pre-Flush1 biofilm iron concentrations, suggesting factors other than iron concentration are influencing discolouration. Given the association between biofilms, discolouration and water quality^5^, microbiological processes are considered likely to influence the observed differences.

### Biofilm cell quantification

**Fig. 4:**
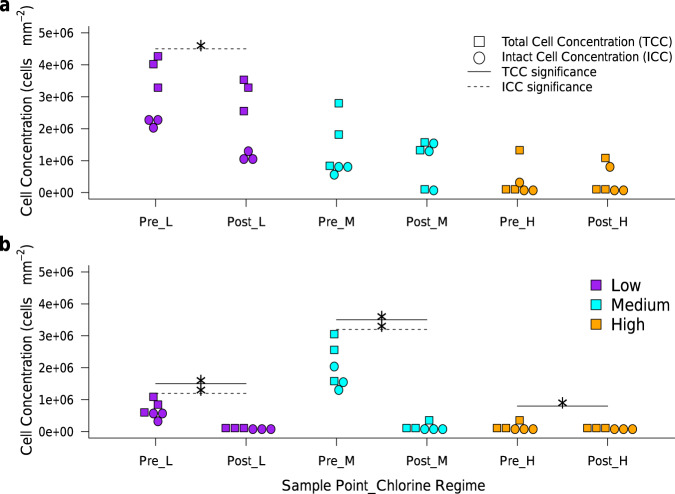
Total and intact cell concentration within DWDS biofilms of each chlorine regime, sampled Pre- and Post-Flush. **a** Test 1, **b** Test 2. L Low-chlorine, M Medium-chlorine, H High-chlorine; Asterisk indicates significant differences between pre- and post-flush biofilms, tested using Wilcox (0 ≤ *W* ≤ 9, 0.04 ≤ *p* ≤ 0.05); where no asterix is shown differences were not statistically significant (2 ≤ *W* ≤ 7, 0.20 ≤ *p* ≤ 0.80).

Irrespective of chlorine regime, biofilm total and intact cell concentrations (TCC and ICC) reduced during flushing (Fig. 4). Application of flow cytometry to DWDS biofilms and bulk water confirmed the relationship between the microbial phases: greatest biofilm TCC and ICC mobilisation was from Low-chlorine, which had the greatest increase of planktonic TCC and ICC, and least discolouration response (Figs. 2 and 4).

In both test 1 and test 2, Pre-Flush biofilms from each regime had significantly different TCC and ICC (Fig. 4; *χ*^2^ ≥ 7, *p* ≤ 0.04) such that High chlorine concentration reduced (but did not prevent) biofilm formation. The cell concentrations observed, particularly in the Low-chlorine biofilms, are higher than previously reported for simulated DWDS with disinfectant residuals^18^ and similar to non-flexible premise plumbing materials tested in a non-chlorinated system^34^. This could be due to variations in methodology, specifically different biofilm removal or homogenisation methods between studies (the analysis herein included singlet-doublet evaluation to ensure homogenisation of ≥98%, see Methods section: cell concentration analysis). Alternatively, this elevated growth may be a consequence of de-chlorinating a previously chlorinated water supply with relatively high organics compared to non-chlorinated DWDS (where maintaining water biostability relies on improved efficiency of organic removal at treatment works), indeed the planktonic cell counts also increased in the Low-chlorine regime (Supplementary Table 1). Previous studies have also shown disinfectant residuals do not prevent biofilm formation^35,36^; at best reducing bacterial concentration^16,18^, biological activity or growth^22,37,38^ crucially, this inhibitory effect does not necessarily apply to other taxa such as fungi^16^. Chlorine residuals may indirectly reduce biofilm cell quantities by reducing the planktonic cells available to colonise the pipe wall (Supplementary Table 1). Intriguingly, increasing residual chlorine did not appear to “kill” biofilm cells at a greater rate; the proportion of ICC (as a % of TCC) was similar between regimes (*χ*^2^ ≥ 1, *p* ≥ 0.15). Potentially, ICC proportions within DWDS biofilms are governed by a constraint other than chlorine (e.g. hydraulics, nutrients, EPS characteristics).

Biofilm cell accumulation and mobilisation were affected by chlorine concentration and inversely correlated with biofilm iron concentration (Figs. 3 and 4; Supplementary Table 2) and water quality degradation (observed as discolouration; Fig. 2, Spearman’s rank correlation could not be calculated reliably for the flushing phases as difference in averages would have had to be used). Note that in test 2 there was an initial and brief lag (<24 h) in reducing chlorine concentrations of the Low-chlorine regime to concentrations comparable to test 1 (Supplementary Fig. 1), this was due to the influx of a fresh water volume after the flushing, which was then dechlorinated. This could have impacted the initial recovery of the Low-chlorine biofilms and led to a decrease in TCC between Post-Flush1 and the Pre-flush2 sample points. Although, other aspects of biofilm behaviour such as succession, community function or the EPS will have likely had a greater impact on biofilm regrowth^16^. In all regimes, EPS accounted for the majority of the biofilm volume that accumulated during growth, not cells (Pre-Flush1 and Pre-Flush2 EPS-to-cell ratios >1; Supplementary Table 3). Also, more EPS than cells were mobilised during flushing (EPS-to-cell ratios decrease; Supplementary Table 3), demonstrating clearly that EPS is an essential biofilm component to consider.

### EPS characterisation

EPS (predominantly comprising proteins and carbohydrates) is produced at an energetic cost to microorganisms for various vital roles^21^, including concentrating (in)organics from the fluid-phase, mechanical stability and disinfection protection. At Pre-Flush1, Low-chlorine biofilms had the most EPS-per-cell (4.39 Arbitrary Units; AU), the presence of residual chlorine reduced EPS-to-cell ratios although no concentration effect occurred between Medium- (1.14 AU) and High-chlorine (1.24 AU; Supplementary Table 3). By Pre-Flush2, High-chlorine and Low-chlorine biofilms had similar EPS-to-cell-ratios. Possibly, EPS production was accelerated during biofilm regrowth in High-chlorine (compared to the growth phase of test 1) due to chlorine-tolerant EPS or microorganisms remaining post-flush, providing a niche and/or community that promoted recolonisation and EPS synthesis.

EPS matrices were generally protein dominated. The EPS of High-chlorine biofilms sampled at Pre-Flush1 had the greatest carbohydrate proportion of all the chlorine regimes, although the matrix was still predominantly protein (Supplementary Table 3). By the Pre-Flush2 sample point, carbohydrates dominated High-chlorine EPS. Chlorine was documented to degrade the EPS of batch-cultured bacterial biofilms, promoting cell survival and culturability^22^. Similarly, chloramine has been shown to reduce the biomass (determined via cell and polysaccharide quantification) of drinking water biofilms in reactors^38^. A study by Xue et al.^39^, found that EPS from *Pseudomonas aeruginosa* biofilms had a high reactivity with chlorine but a low reactivity with monochloramine, this suggests that different protective mechanisms may be inferred by different EPS compositions. High-chlorine biofilms may have synthesised greater protein volumes than other regimes with the elevated residual chlorine “eroding” a proportion, altering the matrix compositional ratio (potentially affecting cohesion/adhesion, viscosity and diffusional properties) and, indirectly, protecting biofilm microorganisms (hence similar biofilm ICC proportions between regimes).

Frequently, proteins were more readily mobilised than carbohydrates: carbohydrate-to-protein ratios increased between biofilms from Pre-/Post-Flush1 and Pre-/Post-Flush2 (Supplementary Table 3). Significant decreases in protein volume (after flushing) were detected where discolouration was the greatest: Medium-chlorine (*W* = 201, *p* < 0.001) and High-chlorine (*W* = 169, *p* < 0.020). Proteins may be more reactive than carbohydrates, with greater influence in concentrating (in)organics (or binding/deactivating chlorine residuals). Subsequently, greater protein mobilisation could release greater metal concentrations, causing elevated discolouration. However, protein mobilisation was not detected from Low-chlorine biofilms, yet a discolouration response was observed. This could be attributed to limits of detection, data variability or scale differences: EPS analysis is based on fields of view, discolouration assesses material mobilised from the entire pipe surface.

Overall, High-chlorine biofilms required the most disinfection protection, potentially conveyed by a different EPS composition, which, indirectly, influenced the concentration of discolouration material. The EPS response to residual chlorine was complex and likely impacted by biofilm development rate, cell growth/replication, nutrient availability, flushing and the ecology of the microbiome synthesising the EPS matrix.

### Biofilm microbiome

**Fig. 5:**
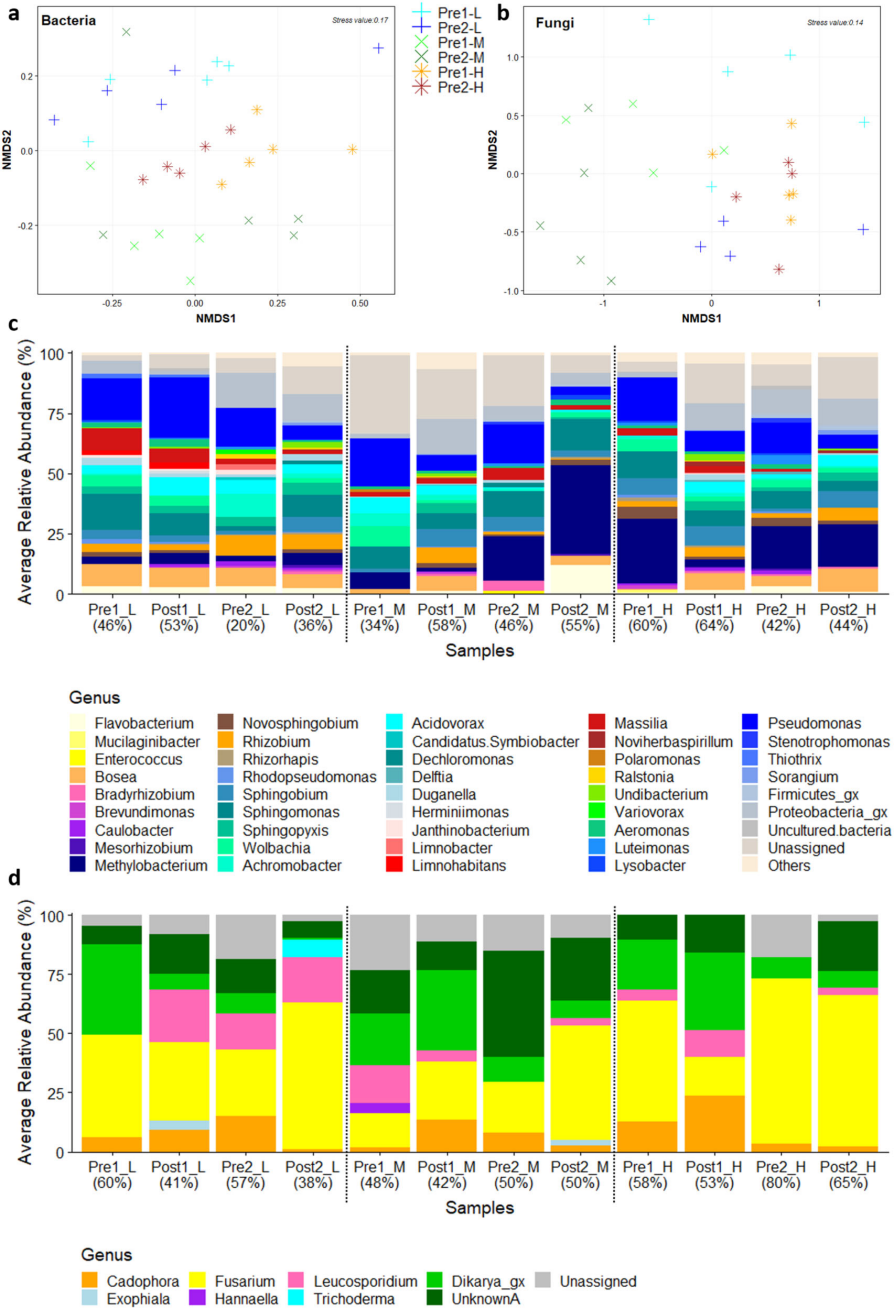
Variation in bacterial and fungal communities of pre- and post-flush biofilm from Low-, Medium- and High-chlorine regimes. nMDS plots based on Bray-Curtis similarities of pre-flush biofilm **a** 16S rRNA and **b** ITS mOTUs. Average relative abundance of **c** bacterial and **d** fungal genera, percentage similarity between bio-replicates (*n* = 5, or *n* = 4, see Methods section: Biofilm microbiome) is shown in brackets, _gx= genus unknown; **c** “Others” ≤1% total relative abundance (Supplementary Table 5); **d** UnknownA=Fungi, further taxonomic information unavailable. Pre1/Pre2 = Pre-Flush1 or Pre-Flush2, Post1/Post2 = Post-Flush1 or Post-Flush 2, L Low-chlorine, M Medium-chlorine, H High chlorine.

Bacterial and fungal communities were distinct between the three chlorine regimes prior to flushing (Fig. 5; ANOSIM, bacteria, global-R = 0.383, *p* < 0.001; fungi, global-R = 0.444, *p* < 0.001) and remained distinct after flushing (Supplementary Fig. 4; ANOSIM, bacteria, global-R = 0.513, *p* < 0.001; fungi, global-R = 0.319, *p* < 0.001). Although, ecological indices (Supplementary Table 4) were unaffected by flushing (or chlorine regime) and had substantial variance, suggesting heterogenic biofilm communities developed. Contrasting trends regarding disinfectant and ecological indices are reported in the literature with some studies reporting decreased biofilm diversity where a chloramine residual was present^13,14^, others an increase in diversity with increased residual concentration^40^. The differing trends suggest that constraints other than oxidative stress are governing diversity; the chloramine studies in particular compared DWDS of different countries with different water sources, treatment and quality, which will have an impact on the microbiome. However, ecological indices are reductive and can only evidence major differences between community structures, the findings in the current study (which benefitted from using the same water source between experiments) are indicative that changes in the biofilm microbiome between chlorine regimes were more subtle. Disinfection is known to alter planktonic bacterial community compositions^10–12,19^ and has previously been demonstrated to also shape the succession of biofilm bacterial communities during growth (there was less of an impact of chlorine on fungi) within the DWDS test facility used in this study, even when the inoculum was pre-conditioned by being chlorinated water^16^. In the current study, pre-flush and post-flush data were analysed (Fig. 5 and Supplementary Fig. 5) to determine any differences in response to flushing between regimes, comparing changes in the biofilm microbiome (structure and composition) that remains adhered, whilst considering the microbial consortium that was mobilised and its impacts on microbial water quality. Consideration is also given to the any compositional differences between regimes at the pre-flush sample points, which could provide insight into the differences in the previously discussed distinct biofilm characteristics (iron concentration and EPS).

A significant shift in bacterial communities was observed during flushing of Medium-chlorine (global-R ≥ 0.384, *p* ≤ 0.016; Fig. 5; and Supplementary Fig. 5) and High-chlorine biofilms (global-R ≥ 0.600, *p* ≤ 0.008; Fig. 5; and Supplementary Fig. 5), where greater discolouration was observed. This indicates the preferential loss of some bacteria over others, such that relative abundances were significantly affected. Conversely, the Low-chlorine pre- and post-flush bacterial communities did not cluster independently and their composition was not significantly affected by flushing (global-R ≤ 0.016, *p* ≥ 0.413; Fig. 5; and Supplementary Fig. 5), perhaps suggesting that there was no-preferential loss of certain bacteria and all taxa were equally likely to be mobilised. The bacterial genera that were highlighted as responsible for driving the differences between pre- and post-flush biofilms were inconsistent between the Medium- and High-chlorine regimes, demonstrating that there was no one key organism that was associated with weakly adhered biofilm and always removed entirely, and rather, a mixed bacterial consortium was mobilised. The mechanical resistance of a biofilm has been described to be governed by the EPS adhesive/cohesive forces, which is perhaps more influenced by environmental parameters than the microbiome, as has been suggested with regard to the impact of hydraulics on DWDS biofilms^41^.

The impact on water quality of the biofilm microbiota being mobilised depends upon the presence and concentration of deleterious microorganisms (aesthetic or potentially pathogenic) in the community that develops and is subsequently detached. At higher taxonomic levels (phyla/class/order) the same bacterial constituents were present in all biofilms prior to flushing (Supplementary Fig. 6), although the dominant taxa varied. However, chlorine regime significantly impacted the presence/absence (global-R ≥ 0.136, *p* ≤ 0.007) and relative abundance (global-R ≥ 0.227, *p* ≤ 0.001) of bacterial families (Supplementary Fig. 4) and genera (Fig. 5). *Alphaproteobacteria* abundance increased with increasing residual chlorine concentration, whilst *Betaproteobacteria* (recently reclassified as the order *Betaproteobacteriales* within *Gammaproteobacteria*^42^) dominated at low chlorine, supporting trends reported at low chloramine concentrations^43^ and those highlighted during detailed analysis of community development in our previous paper^16^. Some phyla such as *Firmicutes* and *Actinobacteria* were absent from Low-chlorine, and some taxa such as *Clostridiaceae* (a family within *Firmicutes*, which includes pathogens) were unique to High-chlorine (Supplementary Fig. 6). This is consistent with trends reported in comparison of chlorinated operational DWDS, where an enrichment of *Firmicutes* was associated with higher residual chlorine concentrations^40^. *Actinobacteria* have been associated with discolouration in field-studies^29^ and reported to dominate under high chloramine^43^, suggesting disinfection/oxidant resilience. Chloramines are reportedly less reactive than chlorine, penetrate biofilms more deeply and persist throughout DWDS^44^. Consequently, the selective pressure of chloramines upon biofilms may be greater than those of free chlorine and the effects observed here perhaps accentuated. Indeed, several studies of chloraminated systems have reported significant enrichment of bacteria that are associated with disinfection tolerance and ammonia oxidation (driven by the input of ammonia into systems due to chloramination)^13,18,38^.

At the genus level, *Pseudomonas, Sphingobium* and *Acidovorax* are commonly detected in water^10,45,46^ and exhibited inconsistent trends with residual chlorine and flushing. This is consistent with previous disinfectant studies, which showed that while there was a selective pressure of disinfectant residual for specific bacteria or genes^13,20,40,38^, there were also shared taxa between biofilms from DWDS with different disinfectant types^20^ or disinfectant concentrations^13^. However, the abundance of some genera (e.g. *Achromobacter*, *Bosea* and *Rhizobium)* decreased with increasing chlorine concentration, while others such as *Novosphingobium, Sphingopyxis* and *Methylobacterium* increased in abundance. Prior to flushing, some bacteria were only detected in one regime, for instance *Rhodobacter*, which at pre-flush was unique to High-chlorine biofilms but then absent from the post-flush High-chlorine samples (Fig. 5). *Methylobacteria* are commonly isolated from drinking water; they are reported to exhibit resistance to disinfectant/cleaning agents^13,40,47,48^, promote aggregation^46,49^ and impact microbially-influenced-corrosion^50^. Possibly the genetic components implicating *Methylobacteria* in microbially influenced corrosion could increase the tendency of High-chlorine bacterial communities to oxidise iron (especially if shared via horizontal gene transfer). Similarly, the exclusivity of *Rhodobacter* (known iron oxidisers^51,52^) to High-chlorine biofilms could help explain the increased iron concentration observed, despite all the regimes being supplied with the same bulk water iron concentrations. Ultimately, despite a greater mobilisation of cells from the Low-chlorine regime (Fig. 4), the absence or reduced abundance of certain bacteria within these communities prior to flushing suggests that the mobilised cells were less associated with iron (and therefore discolouration) than in the Medium- or High-chlorine regimes.

Fungal community compositions generally showed no significant differences due to flushing (global-R ≤ 0.350, *p* ≥ 0.071), apart from between Pre-Flush1 and Post-Flush1 of the Low-chlorine regime (global-R = 0.436, *p* = 0.016; Fig. 5). Nor were there clear trends with chlorine concentration (Fig. 5 and Supplementary Fig. 7), although the communities were distinct between regimes, a finding first reported in Fish and Boxall^16^, which concludes that this is possibly because fungi have greater chlorine tolerance due to their robust morphology^19^. Variation in fungal communities may be stochastic, impacted by the seeding population dictated by upstream environmental pressures, or indicative of an ecological pressure other than chlorine governing taxonomic composition. Despite the contribution that fungi make to the DWDS microbiome they are rarely sampled or monitored in operational DWDS. However, the greater resilience to disinfection and mechanical stress of fungal communities, compared to bacteria, suggests an integral role in biofilm maturation, possibly promoting biofilms that are resistant to cleaning and management practices.

Taxonomic variation in the biofilm communities suggests the cells mobilised from each regime presented different impacts on microbial and aesthetic water quality (and potentially public health). For example, High-chlorine biofilms accumulated fewer cells and lost fewer during flushing, yet they were more likely the source of iron-associated and chlorine-resistant microorganisms, which would be less affected by bulk water chlorine residuals, thus more likely to reach a customer intact. Additionally, studies have reported associations between chlorine or chloramine tolerance and potentially deleterious traits such as antimicrobial resistance^10,17,18,38,53^ or (potentially) pathogenic taxa^14,20,38^, even when biofilm biomass (evaluated by quantifying cells and polysaccharides) was observed to be suppressed by the presence of a residual^38^. If this association is confirmed to be due to the specific selective pressure of disinfection concentration then biofilms such as the High-chlorine biofilms could be an upstream source of antimicrobial resistance genes. For instance, the family *Sphingomonadaceae* have been associated with antibiotic resistance^54^. In the current study, *Novosphingobium, Sphingopyxis* (members of this family) were more abundant in Medium- and High-chlorine regimes than the Low-regime, consistent with trends reported in chlorinated^17^ and chloraminated^38^ systems where *Sphingomonadaceae* taxa increased in abundance with increased disinfectant concentration. With respect to potential pathogens, *Mycobacterium* (a genus which includes opportunistic pathogens) were more abundant in the biofilms of a system with high chloramine concentration (3.8 ± 0.1 mgL^−1^) than a system with no residual (0.08 ± 0.01 mgL^−1^), despite being present at similar concentrations in the bulk water of each system^14^. *Mycobacterium* were not detected in any of the biofilms from the present study, perhaps because of the high abundance of Methylobacteria (the two taxa have been reported to occupy a similar ecological niche^13^.) Conversely, planktonic and biofilm *Legionella* concentrations were reduced by the presence of a disinfectant residual within water mains^15^. These findings, and possible implications, could suggest that an alternative residual disinfection strategy to continuous dosing, such as pulses at high concentrations or cycling concentrations, might be more efficient at harnessing the benefits of disinfection, limiting the impacts of biofilm on water quality whilst reducing the likelihood of selection pressures conditioning for biofilms that are more difficult to manage.

Metal oxides may convey protection to microorganisms by reacting with chlorine residuals and forming deposits^55^. Greater iron accumulation in High-chlorine biofilms may have occurred due to different EPS bio-chemical compositions and the increased occurrence of iron-associated bacteria. Simultaneous increases in chlorine residual protection and discolouration potential (if mobilised) are consequences of this elevated iron. Conversely, Low-chlorine biofilms did not require disinfectant-resistant adaptations, retained greater mechanical stability (with an EPS with a lower propensity to concentrate iron) and caused less discolouration. Considering the biofilm analyses holistically, it is possible that a trade-off (or progression) exists between a biofilm being “chemically” or “mechanically” stable, with adaptations to survive higher chlorine residuals coming at the expense of the ability to resist hydraulic shear.

## Concluding discussion

Environmental stressors are known to govern biofilm formation in aqueous environments. This study provides a rare insight into a critical example of this; biofilm responses to free chlorine residual concentrations in an environment fully representative of HDPE pipes in drinking water distribution systems (DWDS), and their impact upon water quality. The findings indicate that complex selective pressures are placed upon various biofilm characteristics (physical, inorganic and organic) by free chlorine residuals.

This paper presents the first conclusive evidence of increased discolouration (and associated water quality) due to a higher free chlorine residual. While fewer cells were present in, and mobilised from, High-chlorine biofilms, they had the greatest discolouration response with higher metallic concentrations. High-chlorine biofilms had a distinct microbiome, posing a potentially increased contamination impact on microbial water quality than other regimes as they included bacteria that are more likely to be iron-associated and tolerant to oxidants so less likely to be killed/inactivated by the free chlorine residual. Indeed, previous analysis of the biofilm regrowth suggests that a higher free chlorine residual selects for chlorine-tolerant biofilms that are able to recolonise more rapidly^16^. The trends observed pertaining to the High-chlorine regime suggest that continually maintaining a higher residual concentration could condition for biofilms that present a continuing threat to water quality due to the microbial consortium that could be mobilised, the difficulties in cleaning/managing the associated elevated inorganic accumulation and the potential for any mobilised material to persist in the bulk water (by retaining a coating of chlorine-resistant EPS). Of note is that the increased cell and EPS quantity in Low-chlorine biofilms did not translate to greater water quality degradation or water quality failures (with respect to discolouration or microbial content).

An extrapolation of this study could be the reduction or elimination of free chlorine residuals to reduce the scale of a discolouration response and chance of a quality failure, particularly when considered in combination with the risks associated with emerging disinfection by-products and environmental impacts of chemical use. As chlorine residual use is not a regulatory requirement, this is not impractical. The World Health Organisation (WHO) states in its Guidelines for Drinking Water Quality that it may be “appropriate to add a small dose of a persistent disinfection such as chlorine or monochloramine to act as a preservative during distribution”^56^. WHO do not state that secondary chlorination (i.e. following primary disinfection within water treatment) is a specific requirement prior to distribution, and residual disinfection legislation is absent from EU Drinking Water Directives. However, the benefits of chlorine residuals in minimising regrowth and mitigating microbial contamination in ageing DWDS must not be ignored. Countries not employing residuals have younger DWDS assets, lower leakage and achieve higher quality treated water which is more bio-stable. Hence it is vital that residual concentrations are set and managed considering all risks to water quality and as part of integrated strategies.

Biofilms are dynamic, adapting to our management of environments in complex ways. The integrated physical, chemical and biological analysis conducted here revealed the complexity of the causative processes behind the selective pressure of free chlorine residual on DWDS biofilms. This research demonstrates that such holistic analysis and interpretation is essential to understanding the consequences of, and hence inform, management of any environment. Consideration of such interactions is critical for improving disinfection strategies to manage biofilms (or biofouling) and specifically offers understanding within a DWDS context, to mitigate unintended impacts of residual chlorine, protecting future water quality compliance and hence safety.

## Methods

### DWDS experimental facility

The full scale, temperature controlled, drinking water distribution system (DWDS) experimental facility at the University of Sheffield (department of Civil and Structural Engineering) comprises ~610 m length of high density polyethylene (HDPE PE100) pipe configured to run as three independent loops (Fig. 1a). All tests were conducted at 16 °C, representative of U.K. summer water temperatures when discolouration events are most common. Each loop is 203 m in length, predominantly 79.3 mm internal diameter (flow metres and control valves were installed in a short section of 50 mm internal diameter to allow for improved flow control and monitoring accuracy; Fig. 1). The large pipe length ensures effects are dominated by in-pipe processes. Each loop was connected to its own enclosed reservoir tank and pump, the tanks are supplied from the same inlet, with water direct from the trunk main of the local DWDS (high organics upland catchment, ferric based treatment and cast iron trunk mains). Water was recirculated around the loops following a diurnal flow pattern (Supplementary Fig. 1) typical of residential DWDS, with an independent system residence time of 24 h, controlled with a trickle drain/fill (Fig. 1). This provided common baseline water quality (e.g. the supply of nutrients, metals, other inorganics, disinfectant and microorganisms) for all loops, determined by the local water supply. Online instrumentation in each loop measured free chlorine (ATi Q45H Free Chlorine Monitor, Analytical Technology Inc., UK), turbidity (ATi A15/76 Turbidity Monitor, Analytical Technology Inc., UK) and flow rate (Mag X2 flow meter, Arkon Flow Systems, Czech Republic). Additionally, each loop had two straight sections with 27 apertures (54 in total per loop) located around the circumference of the pipeline into which Pennine Water Group (PWG) coupons^25^ were inserted providing a removable surface for sampling biofilms (Fig. 1). It has previously been ascertained that biofilm structure was unaffected by the position around the pipe circumference^57^, therefore, samples were not separated on this basis for the analysis presented herein (although a combination of crown, invert and side samples were taken at each sample point). Note that PWG coupons comprise an outer coupon and a removable insert as shown, which enabled dual analysis of the same sample.

### Chlorine regimes and biofilm development

A 28-day biofilm accumulation period was selected as indicative of initial colonisation of replaced/relined pipes, additionally previous studies demonstrated this timeframe generated measurable responses during flushing, with respect to turbidity and biofilm^24,58^. During growth the flow in each loop was controlled to follow the same common double-peaked diurnal residential demand pattern (Supplementary Fig. 1).

Biofilms were developed naturally (i.e. no artificial seeding) under one of three chlorine regimes (Supplementary Fig. 1) referred to as: High-, Medium-, or Low-chlorine. Briefly, Medium-chlorine concentration was determined by the incoming water, system retention time and any chlorine demand of the facility, hence experienced natural variations throughout growth. Residual chlorine concentrations were boosted by 0.4–0.5 mgL^−1^ in the High-chlorine regime, and incoming water was dechlorinated in the Low-chlorine regime, by constant drip-dosing with a 1:15 (v/v) dilution of 12% sodium hypochlorite or a 1% sodium ascorbate solution (Vit-D Chlor, USA), respectively. The target High-chlorine concentration increase was selected (based on preliminary tests) to be significantly different from the Medium-chlorine and ensure final average residual chlorine concentrations did not exceed 1 mgL^−1^, the top level targeted in most U.K. systems. Dosing solutions were protected from light, supplied into the tank (at the point of the trickle feed) of the appropriate loop, via a peristaltic pump and changed every three days. High- and Low-regimes were dosed at consistent rates and thus experienced identical natural variations in chlorine concentration to the control but at higher or lower concentrations (Supplementary Fig. 2). Note that for the growth phase of test 2, the Medium- and High-chlorine regimes were started a day later than the Low-chlorine regime due to the time required to conduct the initial flushing phase of the three systems in test 1 prior to starting test 2.

Throughout the growth periods free chlorine, turbidity and flow-rate were recorded continuously using the aforementioned online equipment (see “DWDS experimental facility”), other water quality parameters were monitored via spot samples (see Supplementary Table 1). Total organic carbon (TOC) was monitored during preliminary tests and did not differ between regimes (average concentration of 1.64 mg L^−1^).

### Flushing and discolouration response

After growth, a flushing phase was conducted for test 1 and test 2. This required water to be sealed within each loop and tank (dosing and trickle feed/drain stopped). Each loop was independently flushed at four increasing flow rates (0.74, 3.58, 5.10 and 6.29 ls^−1^) to increase the shear stress (the force perpendicular to the pipe wall) incrementally (0.09, 1.57, 3.05 and 4.53 Pa), within ranges observed in operational networks during routine maintenance or following hydraulic events. Each flow rate was sustained for five turnovers to ensure thorough mobilisation and mixing over the entire pipe length/water volume. The initial flushing step (0.09 Pa) was a mixing phase, during which background water quality data was collected. Water quality data from the subsequent flow rate/shear stress steps was normalised against this mixing step to determine any changes in parameters due to the flushing process.

During flushing the flow rate, turbidity and chlorine were monitored continuously using the aforementioned online instruments (see “DWDS experimental facility”). Note that measurements were taken every second, hence the replication for turbidity (Fig. 2) ranged from *n* = 505 to *n* = 5470, decreasing with increasing flow rate as the time to complete five turnovers reduced. Additionally, the water quality parameters in Supplementary Table 1 were measured (spot samples, *n* = 3) after one turnover of each shear stress increment to detect any initial material mobilisation into the water column before dilution during the subsequent four turn overs.

Each loop was flushed sequentially, biofilm samples were collected just before the flushing to ensure they accurately represented the pre-flush biofilms. Therefore, Pre-Flush1 biofilms (from the end of the test 1 growth phase) were collected over a 27-h period such that High-chlorine was sampled on Day 28, Low- and Medium-chlorine biofilms were sampled on Day 29. Due to the subsequently staggered start of the test 2 growth phase, which was run directly after the flushing of test 1 was completed, all the Pre-Flush2 biofilm samples were collected after an additional 28 days of growth.

### Biofilm sampling

Biofilm samples (*n* = 8 coupons, i.e. inserts and outer sections; Fig. 1) were collected from each chlorine regime at Day 0 of test 1 (controls), the end of the growth phase of test 1 and test 2 (i.e. Pre-Flush1/Pre-Flush2) and after the flushing of test 1 and test 2 (i.e. Post-Flush1/Post-Flush2). Biofilms were compared between regimes with respect to: inorganic content (*n* = 3 outers); total and intact cell concentrations (*n* = 3 inserts); EPS characteristics (*n* = 3 inserts); and bacterial or fungal community compositions (*n* = 5 outers). Where biofilm suspensions are subsequently mentioned, biofilm was removed from the outer/insert using standardised brushing into 30 mL of phosphate buffer solution (PBS), sterile coupons and/or PBS were used as negative controls.

### Biofilm inorganic analysis

Biofilm suspensions (from outer coupons, *n* = 3) were filtered through a 25-mm diameter, 0.2 µm pore nitrocellulose membrane (Millipore) and stored in the dark (at 4 °C) prior to analysis under vacuum using a PANalytical Zetium X-ray fluorescence spectrometer and PANalytical’s proprietary Omnian analysis package to determine the relative concentrations of various elements. These “elemental fingerprints” were normalised by removing the average amount of each element present in controls (membranes with pure PBS filtered through them), oxygen was also removed as this consistently dominated due to elements existing predominantly as oxides. Normalised “inorganic-fingerprints” were analysed via principal-components-analysis (PRIMER-E; Supplementary Fig. 3). PC1 and PC2 explained 87.0–99.8% variation; at Pre-Flush1/Pre-Flush2 iron and chlorine were responsible for most of the variation between regimes (phosphorus and sodium differed but to a lesser extent). Iron was no longer a dominant factor after flushing, chlorine explained most (≥95.9%) of the variation between regimes (Supplementary Fig. 3; phosphorus and sodium importance reduced by a magnitude). Iron and manganese are related to discolouration (and were measured in bulk water during flushing) so their biofilm concentration was of particular relevance to this study. Manganese was only detected in three samples (all from High-chlorine) therefore statistical analysis was not possible (% concentrations were Pre-Flush1 = 0.004, Post-Flush1 = 0.002 and Pre-Flush2 = 0.010). Conversely, iron was detected in all samples from each regime (*n* = 36) so downstream analysis was possible.

### Cell concentration analysis

Total and intact cell concentrations (TCC and ICC) were determined by analysing bulk water (dechlorinated with sodium ascorbate) or biofilm suspensions (from inserts with surface area of 90 mm^2^, homogenised via vortexing) in accordance with the flow cytometry protocol and analysis detailed elsewhere^59,60^. Briefly, 0.5 mL of the sample was stained with either SYBR Green to count total cells, or SYBR Green and Propidium Iodide (Invitrogen) to count intact cells. Samples were incubated (10 minutes, 37 °C) and analysed with a BD Accuri C6 Flow Cytometer (50 µL, medium flow rate). The standard analysis template^59^ was edited to include singlet-doublet analysis, providing a quantitative assessment of sample homogenisation (Supplementary Fig. 8); in all samples (planktonic and biofilm) ≥98% of the data were singlets. Prior to the calculation of averages and statistical tests, the total or intact cell-counts-per µL obtained from the C6 software were converted to TCC or ICC concentrations (mL^−1^ for planktonic—multiply count by 1000 µL; mm^2^ for biofilms – multiply counts by suspension volume of 30 mL, then divide by surface area from which biofilm was removed).

### Biofilm physical structure characterisation

EPS and cells were characterised using confocal laser scanning microscopy (CLSM) and digital image analysis (DIA) as described elsewhere^57^. The CLSM method applied is currently the only robust EPS analysis suitable for these DWDS biofilms. Briefly, samples (inserts, *n* = 5) were fixed (5% formaldehyde, 48 h), washed in PBS and stained with SYTO 63 (targets cells), fluorescein-5-isothiocyanate (FITC) (targets proteins) and Concanavalin-A tetramethylrhodamine (Con-A) (targets carbohydrates). Lambda-Z-stack images were generated for five Fields of View (FOV; 420 µm × 420 µm) per sample, using an LSM510 meta upright CLSM with an x20 EC Plan Neofluar objective (0.5 NA). Autofluorescence was removed using the “unmix” function of LSM510 software (Kroto Imaging Facility, The University of Sheffield, UK). Subsequently, Python v2.7.2 (www.python.org) and R v3.5^61^ were used to apply a median filter (to reduce noise); thresholding (values: SYTO 63 = 1101, FITC = 701 and Con-A = 501) and calculate the volume of each stained component, as well as the EPS ratios^57^, expressed as arbitrary units (AU).

### Biofilm microbiome

Bacterial and fungal biofilm communities were evaluated using the protocol detailed elsewhere^16^. Briefly, biofilm suspensions (*n* = 5) were concentrated via filtering (0.22 µm pore nitrocellulose membrane) and DNA was extracted using the proteinase K chemical lysis method^57,62^. DNA sequencing was conducted at the NERC Biomolecular Analysis Facility at the University of Sheffield, using the Illumina MiSeq platform. Bacterial 16S rRNA genes were amplified using the primers 63F (5′-CAGGCCTAACACATGCAAGTC-3′) and 518R (5′-CGTATTACCGCGGCTGCTCG-3′)^63^. Fungal ITS regions were amplified using ITS1F (5′- CTTGGTCATTTAGAGGAAGTAA-3′) and ITS4 (5′-TCCTCCGCTTATTGATATGC-3′)^64^. Sequencing was successful for most samples resulting in replication of *n* = 5 for downstream analysis for the majority of sample points, for both taxa, apart from *n* = 4 for bacteria Post2-L and Post2-H and fungi Pre1-M, Post1/2-H and Post1/2-M.

Sequence processing followed the previously published protocol^16^, using Trimmomatic^65^, FLASH^66^ with a 5% mismatch threshold, MOTHUR^67^ to extract aligned sequences and USEARCH^68^ to check for (and remove) chimeras and singletons, de-replication and clustering (at 97% identity). Matrices of the presence/absence and relative abundance of molecular Operational Taxonomic Units (mOTUs) within each sample were created using USEARCH and Rv3.5. Two databases were used for classifying the cleaned sequences: SILVA^69^ for bacteria and UNITE^70^ for fungi. Confidence interval was set at 95% and taxa were assigned via the lowest common-ancestor algorithm (MEGAN)^71^.

Data was analysed using the R packages: VEGAN, fossil, ggplot2, RColorBrewer, ggthemes, dendextend and clustsig. Ecological indices calculated were richness (Chao1), diversity (Shannon) and evenness (Simpson-inverted). Non-parametric multidimensional scaling (nMDS) was used to visualise the resemblance between communities (for presence/absences and relative abundance data), based on Bray-Curtis similarities and plotted using ggplot2 (Supplementary Fig. 5 used dendrograms to visualise the similarity data, plotted using dendextend in Rv4). Analyses of similarity (ANOSIM) were calculated to ascertain statistically significant differences in bacterial or fungal communities between chlorine regimes (and sample points), global-R (ranging from 0 to 1, where 0 indicates no difference in communities) and *p*-values are presented.

Average relative abundance of each taxa was calculated for each sample group, at each level (phyla, class, order, family and genus) across the replicates (*n* = 5). Data was standardised for comparison (expressed as % total abundance) and plotted as stacked bar charts (using Rv3.5), including any taxa that accounted for >1% of the total relative abundance in at least one sample. Any taxa ≤1% were collated and termed “Others” (see Supplementary Table 5). SIMPER analysis (Similarity Percentage analysis; PRIMER-E v6) assessed replicate similarity within a sample group and determined the family or genera predominantly driving (threshold of ≥75%) the observed differences between the chlorine regimes.

### Statistical analysis

With respect to all the measured parameters, Pre-Flush1 and Day 0 samples differed due to biofilm development, Day 0 (controls) did not differ between chlorine regimes. These are expected trends, reported numerous times before for biofilm maturation in diverse environments hence details are not presented. Note that throughout the manuscript replication (*n*) refers to the distinct biological replicates which were measured and analysed. Unless otherwise stated, water quality and biofilm parameters were compared between chlorine regimes or sample points using the non-parametric tests Kruskal (df = 2) or Wilcoxon, *χ*^2^ and *W* values are presented, respectively, in addition to *p*-values. Where regression analysis was applied (*R*^2^ and *p*-values are presented), the linear models were compared using analysis of co-variance (ANCOVA) tests (F and *p*-values presented). All statistical tests and plotting of figures were carried out in R v3.5^61^, unless otherwise specified.

### Reporting summary

Further information on research design is available in the Nature Research Reporting Summary linked to this article.

## Supplementary information

Reporting Summary

Supplementary Information

## Data Availability

The authors declare that all the data supporting the findings of this study are available within the paper (and its Supplementary files) and that raw data was presented where possible. The raw MiSeq data reported in the paper (Fig. 5 and Supplementary Figures and Tables) have been uploaded to the NCBI Sequence Read Archive under accession numbers PRJNA655920 and SUB7844933. The other datasets used for Figs. 2–4 and Supplementary Figures and Tables are available via the University of Sheffield open data repository system (ORDA: 10.15131/shef.data.12728603).
