# Author Correction: Uncharted waters: the unintended impacts of residual chlorine on water quality and biofilms

**DOI:** 10.1038/s41522-022-00318-8

**Published:** 2022-07-12

**Authors:** Katherine E. Fish, Nik Reeves-McLaren, Stewart Husband, Joby Boxall

**Affiliations:** 1 Sheffield Water Centre, Department of Civil and Structural Engineering, University of Sheffield, Sheffield, S1 3JD UK; 2 NERC Biomolecular Analysis Facility, Department of Animal and Plant Sciences, University of Sheffield, Western Bank, Sheffield, S10 2TN UK; 3 Department of Materials Science and Engineering, University of Sheffield, Sheffield, S1 3JD UK

**Keywords:** Applied microbiology, Biofilms, Water microbiology, Microbial ecology, Microbiome

Correction to: *npj Biofilms and Microbiomes* 10.1038/s41522-020-00144-w, published online 25 September 2020

The original title of this Article had a typo. This has now been corrected in both the HTML and PDF versions of the Article.

