# Supplementary material for: Uncharted waters: the unintended impacts of residual chlorine on water quality and biofilms
Source: NPJ Biofilms Microbiomes. 2020 Sep 25;6:34. doi: 10.1038/s41522-020-00144-w (PMC7519676; doi:10.1038/s41522-020-00144-w)
Supplement: Supplementary file 1 — Reporting Summary [file 41522_2020_144_MOESM1_ESM.pdf]

nature research

Corresponding author(s): Katherine Fish

Last updated by author(s): Aug 10, 2020

Reporting Summary

Nature Research wishes to improve the reproducibility of the work that we publish. This form provides structure for consistency and transparency in reporting. For further information on Nature Research policies, see our Editorial Policies and the Editorial Policy Checklist.

Statistics

For all statistical analyses, confirm that the following items are present in the figure legend, table legend, main text, or Methods section.

n/a	Confirmed	
☐	☒	The exact sample size (*n*) for each experimental group/condition, given as a discrete number and unit of measurement
☐	☒	A statement on whether measurements were taken from distinct samples or whether the same sample was measured repeatedly
☐	☒	The statistical test(s) used AND whether they are one- or two-sided<br>*Only common tests should be described solely by name; describe more complex techniques in the Methods section.*
☐	☒	A description of all covariates tested
☐	☒	A description of any assumptions or corrections, such as tests of normality and adjustment for multiple comparisons
☐	☒	A full description of the statistical parameters including central tendency (e.g. means) or other basic estimates (e.g. regression coefficient) AND variation (e.g. standard deviation) or associated estimates of uncertainty (e.g. confidence intervals)
☐	☒	For null hypothesis testing, the test statistic (e.g. *F*, *t*, *r*) with confidence intervals, effect sizes, degrees of freedom and *P* value noted<br>*Give P values as exact values whenever suitable.*
☒	☐	For Bayesian analysis, information on the choice of priors and Markov chain Monte Carlo settings
☒	☐	For hierarchical and complex designs, identification of the appropriate level for tests and full reporting of outcomes
☒	☐	Estimates of effect sizes (e.g. Cohen's *d*, Pearson's *r*), indicating how they were calculated

Our web collection on statistics for biologists contains articles on many of the points above.

Software and code

Policy information about availability of computer code

Data collection	Continuous water quality data was collated into .csv files via LABview. Spot sample water quality data was collected by hand and recorded as .csv file. Flow cytometery data was collected using the BD C6 software. Confocal microscopy used the Zeiss LSM510 software provided with the CLSM microscope. For inorganic analysis PANalytical software for the PANalytical Zetium XRF equipment was used.
Data analysis	R v3.5 was used for all statistical testing and plotting, apart from the PCA analysis which was undertaken in PRIMER-E and some of the EPS analysis which used python. Bioinfomatics used the following software: Trimmomatic, FLASH, MOTHUR, USEARCH, MEGAN. Full details are provided in the Methods section of the manuscript.

For manuscripts utilizing custom algorithms or software that are central to the research but not yet described in published literature, software must be made available to editors and reviewers. We strongly encourage code deposition in a community repository (e.g. GitHub). See the Nature Research guidelines for submitting code & software for further information.

Data

Policy information about availability of data

All manuscripts must include a data availability statement. This statement should provide the following information, where applicable:

- Accession codes, unique identifiers, or web links for publicly available datasets
- A list of figures that have associated raw data
- A description of any restrictions on data availability

The authors declare that all the data supporting the findings of this study are available within the paper (and its supplementary files) and that raw data was presented where possible. The raw MiSeq data reported in the paper (Figure 5 and supplementary figures and tables) have been uploaded to the NCBI Sequence Read Archive under accession numbers PRJNA655920 and SUB7844933. The other datasets used for Figures 2-4 and supplementary figures and tables are available via the University of Sheffield open data repository system (ORDA: https://doi.org/10.15131/shef.data.12728603).

Field-specific reporting

Please select the one below that is the best fit for your research. If you are not sure, read the appropriate sections before making your selection.

☐ Life sciences ☐ Behavioural & social sciences ☒ Ecological, evolutionary & environmental sciences

For a reference copy of the document with all sections, see nature.com/documents/nr-reporting-summary-flat.pdf

Ecological, evolutionary & environmental sciences study design

All studies must disclose on these points even when the disclosure is negative.

Study description	This study aimed to ascertain the impacts of residual chlorine concentrations on DWDS biofilm characteristics and discolouration (a water quality issue) response. Specifically, we aimed to establish if the action of free chlorine residuals to suppress planktonic regrowth applied to biofilms, and determine any subsequent (unintended) impacts on water quality. Microbial biofilms were developed for 28 days within a laboratory based full-scale drinking water distribution experimental system, under one of three chlorine regimes - Low, Medium or High, with increasing residual chlorine concentration. This system replicates full-scale conditions, is fed with drinking water from the local network and is temperature controlled, providing samples relevant to the real-world under laboratory level control. After the growth period, the discolouration response of each regime was determined by applying an intervention, termed “flushing”, during which flow rates were increased incrementally (thus elevating shear stress) and water quality was monitored to detect any subsequent changes. The growth and flushing phases were then repeated.
Research sample	Biofilm samples from the pipe wall were obtained, along with water samples, from each of the three chlorine treatments. The biofilm samples were analysed for various physical and community based parameters as detailed in the methods, with respect to taxa bacteria and fungi were amplified from the environmental samples that were obtained.
Sampling strategy	The sampling strategy was determined by the number of coupons (used to obtain the biofilm samples) which were available within the pipe loop test facility. Replication and sampling is detailed in the methods of the manuscript. In brief, biofilms were sampled at Day 0, Pre-flush1, Post-flush1, Pre-flush2 and Post-flush2 with a total of 8 coupons being taken at each time point.
Data collection	Data was collected and analysed by Katherine Fish according to the details provided in the methods.
Timing and spatial scale	The tests were run over a 9 week period in 2015. The repeated tests were run straight after the first tests, wtih samples taken at the start and end of growth and after the flushing as described in detail in the methods of the manuscript. These timepoints were selected to enable comparison of the response of biofilms from different chlorine regimes to the flushing and also enable comparison of the types of biofilms that had developed prior to flushing.
Data exclusions	The only data excluded were outliers which are specifcally mentioned in the manuscript or, for the seqeuncing data, bio-replicates are missing where bacterial or fungal amplicons were unable to be extracted and amplified to detectable levels.
Reproducibility	This experiment was conducted three times: preliminary tests (data provided in supplementary information), the initial growth/flush phase (Day 0, Pre-flsuh1, Post-flush1) and the repeated growth/flush (Pre-flush2, Post-flush 2).
Randomization	Biofilm samples were taken from random points along the pipe loop and from around the entire circumference of the pipe (crown, middle, invert), from each of the three treatments (Low, Medium and High chlorine).
Blinding	Samples were given numbers to avoid any bias during analysis.

Did the study involve field work? ☐ Yes ☒ No

Reporting for specific materials, systems and methods

We require information from authors about some types of materials, experimental systems and methods used in many studies. Here, indicate whether each material, system or method listed is relevant to your study. If you are not sure if a list item applies to your research, read the appropriate section before selecting a response.

Materials & experimental systems

n/a	Involved in the study	
☒	☐	Antibodies
☒	☐	Eukaryotic cell lines
☒	☐	Palaeontology and archaeology
☒	☐	Animals and other organisms
☒	☐	Human research participants
☒	☐	Clinical data
☒	☐	Dual use research of concern

Methods

n/a	Involved in the study	
☒	☐	ChIP-seq
☐	☒	Flow cytometry
☒	☐	MRI-based neuroimaging

Flow Cytometry

Plots

Confirm that:

- [x] The axis labels state the marker and fluorochrome used (e.g. CD4-FITC).
- [x] The axis scales are clearly visible. Include numbers along axes only for bottom left plot of group (a 'group' is an analysis of identical markers).
- [x] All plots are contour plots with outliers or pseudocolor plots.
- [x] A numerical value for number of cells or percentage (with statistics) is provided.

Methodology

Sample preparation	Cells were obtained from drinking water biofilms and stained using Sybr Green and a SybrGreen and Propidium Iodide mix, as detailed in the methods.
Instrument	BD Accuri C6
Software	BD Accuri C6 software.
Cell population abundance	Total and intact cell counts were made on the mixed-taxa biofilm samples by removing the biofilm and making an homogenised suspension which was then analysed using the standard BD C6 gating template with an addition of the time and singlet/doublet plots as shown in supplementary information. Water samples were de-chlorinated and then stained and run using the above procedure.
Gating strategy	The tandard BD C6 gating template with an addition of the time and singlet/doublet plots as shown in supplementary information.

- [x] Tick this box to confirm that a figure exemplifying the gating strategy is provided in the Supplementary Information.
