# Supplementary material for: Uncharted waters: the unintended impacts of residual chlorine on water quality and biofilms
Source: NPJ Biofilms Microbiomes. 2020 Sep 25;6:34. doi: 10.1038/s41522-020-00144-w (PMC7519676; doi:10.1038/s41522-020-00144-w)
Supplement: Supplementary file 2 — Supplementary Information [file 41522_2020_144_MOESM2_ESM.pdf]

**Supporting Information for "Uncharted waters: the unintended impacts of residual-chlorine on water quality and biofilms"**

*Katherine E. Fish*, Nik Reeves-McLaren, Stewart Husband, Joby Boxall*

*(*Corresponding Author: Katherine Fish)*

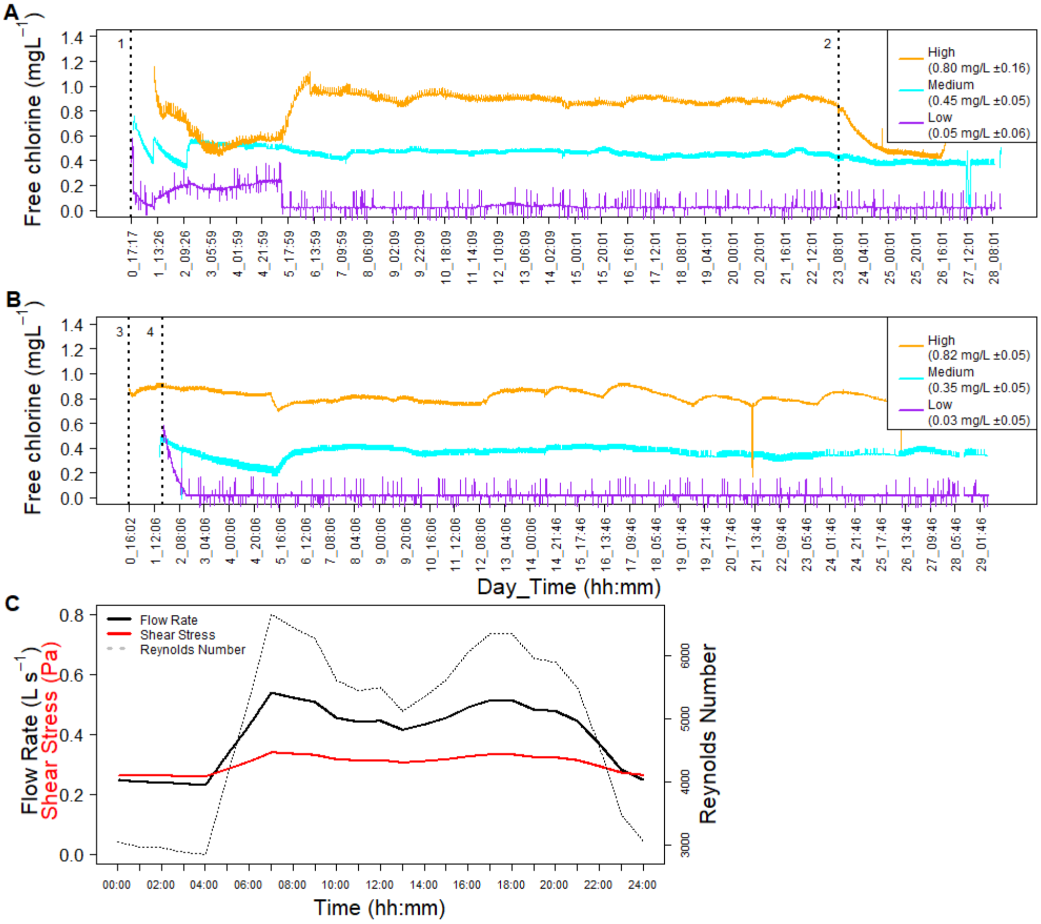


**Supplementary Figure 1 Chlorine and hydraulics during biofilm growth phases.** Residual free chlorine concentration for (A) Test 1 and (B) Test 2, raw data is plotted (n≥39986), the average concentration (± standard deviation) stated. 1, 3 & 4=dosing started in High- or Low-chlorine regimes, 4 indicates staggered start of Test 2. 2=Dosing interrupted in High-chlorine for ~48 hours. (C) Diurnal flow profile used with an average flow rate of 0.4ls$^{-1}$ (0.30 Pa), the average flow rate in UK DWDS for 75-100 mm diameter pipes;[52] low-flow of 0.23ls$^{-1}$ (0.25 Pa), peak-flow of 0.54 ls$^{-1}$ (0.34 Pa).

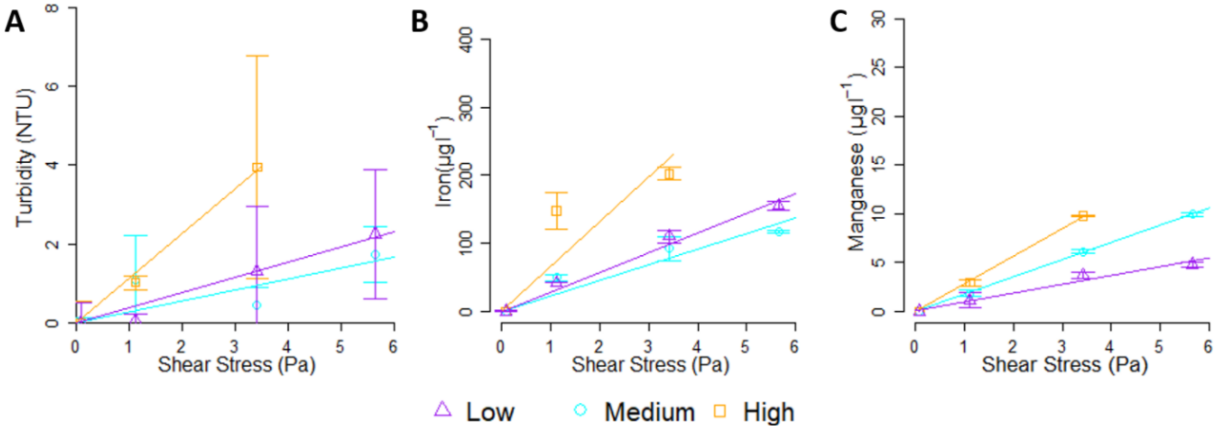


**Supplementary Figure 2 Response of water quality discolouration parameters to elevations in shear stress.** A) Turbidity; B) Iron concentration; C) Manganese concentration during preliminary tests of the three chlorine regimes Low, Medium, High. Averages (n=3) ± standard deviation are plotted, final shear stress of the High-chlorine flushing was unable to be sampled due to technical issues.

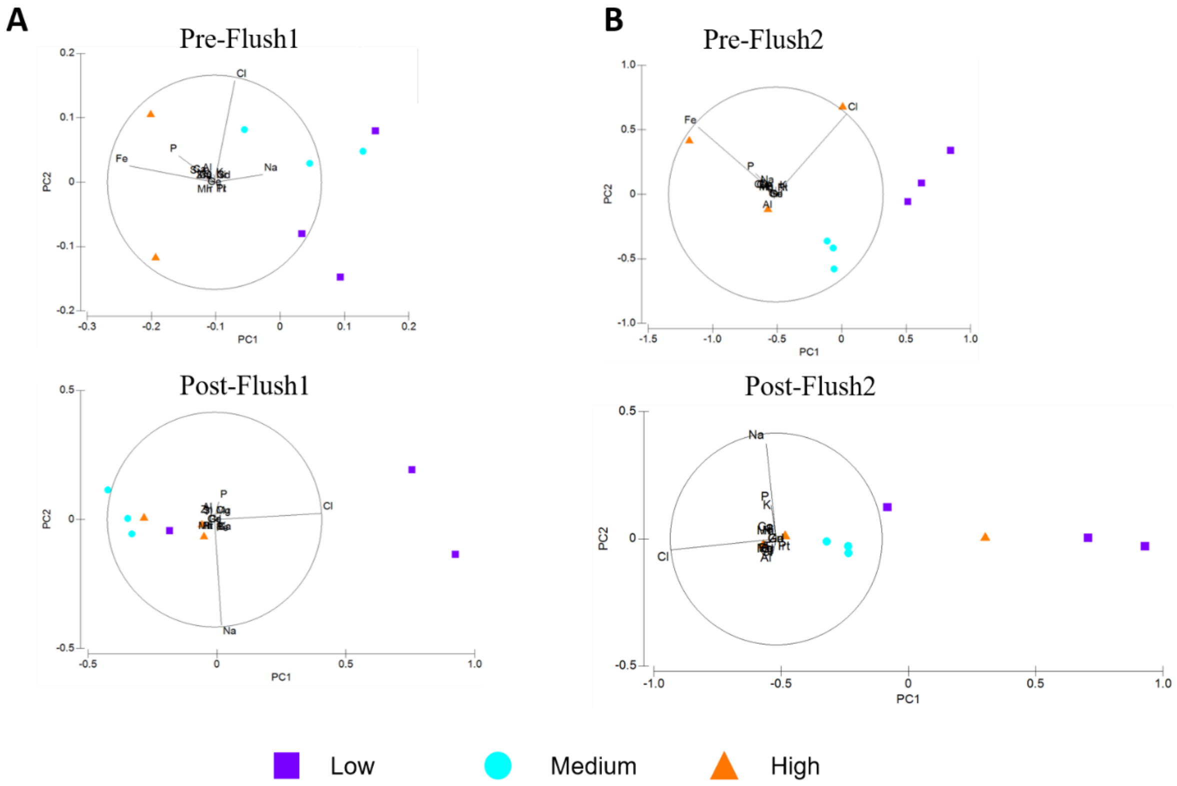


**Supplementary Figure 3 PCA analysis of biofilm elemental fingerprints from Low-, Medium- and High-chlorine regimes before and after flushing for A) Test 1; B) Test 2.** n=9, samples are coloured according to chlorine regime as indicated. Elements included: Al, Ca, Cl, Cr, Cu, Fe, Gd, Ge, Ir, K, Mg, Mn, Na, Ni, P, Pt, S, Si, Ti, Zn. Oxygen was removed during the normalisation process, all profiles were normalized to control samples (n=3). Chlorine regimes were distinct at all time points (assessed via ANOSIM) but were less similar before flushing (global-R≥0.456, p≤0.046) than after (global-R≤0.358, p≤0.043).

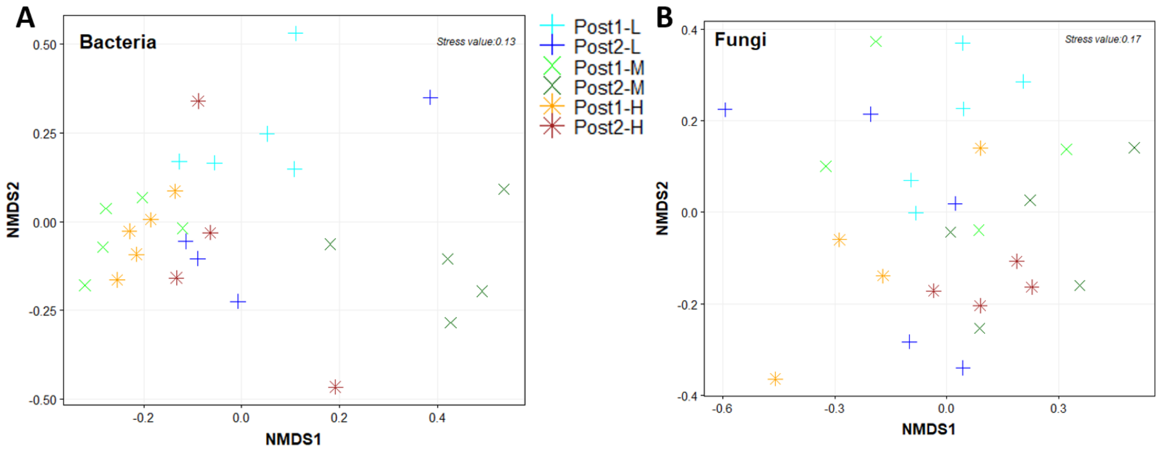


**Supplementary Figure 4 Variation in bacterial and fungal communities of the post-flush biofilms from Low-, Medium- and High-chlorine regimes.** nMDS plots based on Bray-Curtis similarities of post-flush biofilm (A) 16S rRNA and (B) ITS mOTUs. Post1 or Post2 indicates Post-Flush1 or Post-Flush 2 samples, L=Low-chlorine, M=Medium-chlorine, H=High chlorine.

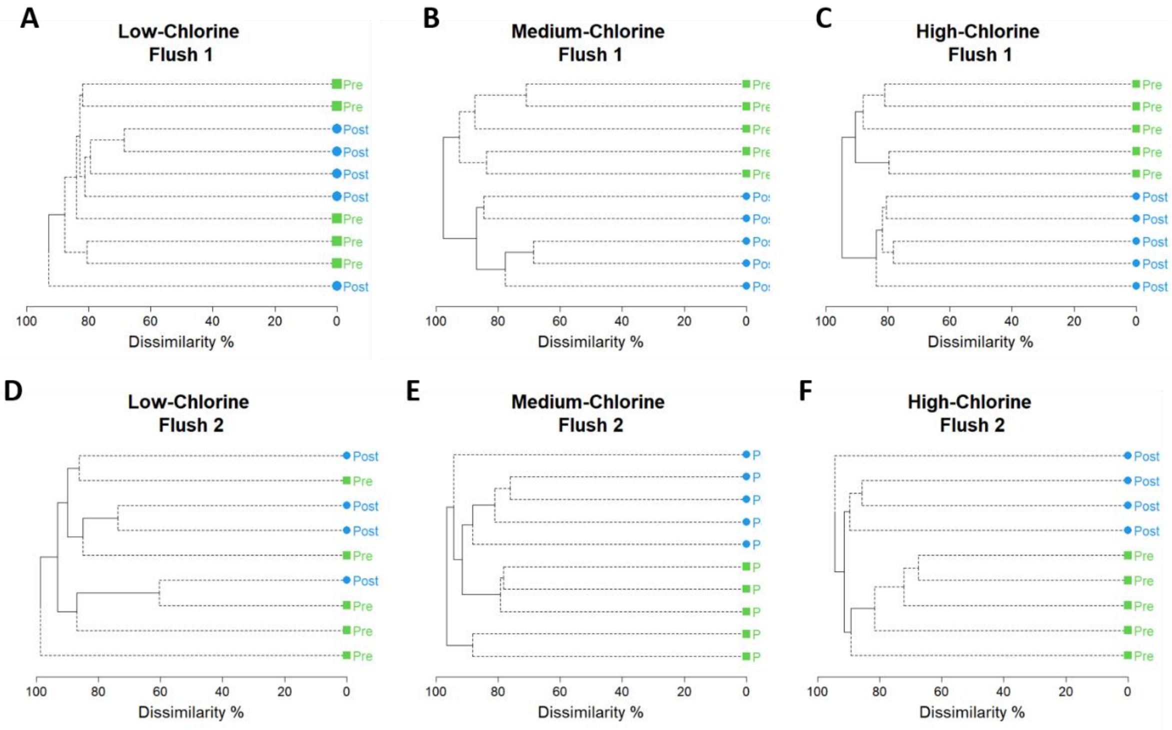


**Supplementary Figure 5 Cluster analysis to show the dissimilarity between bacterial communities in pre- and post-flush biofilms from (A/D) Low-, (B/E) Medium- and (C/F) High-chlorine regimes.** Dashed lines indicate samples which were not significantly dissimilar according to SIMPROF. Green squares and labels indicate pre-flush biofilms, blue circles and labels indicate post-flush biofilms, the chlorine regime and flush (1 =A-C, 2=D-F) are indicated above each dendrogram. Dendrograms were plotted using seqeuncing data at the mOTU level. Data was square root transformed, a resemblance matrix was generated via a Bray-Curtis dissimilarity test and hiarachical clustering using group averages was applied.

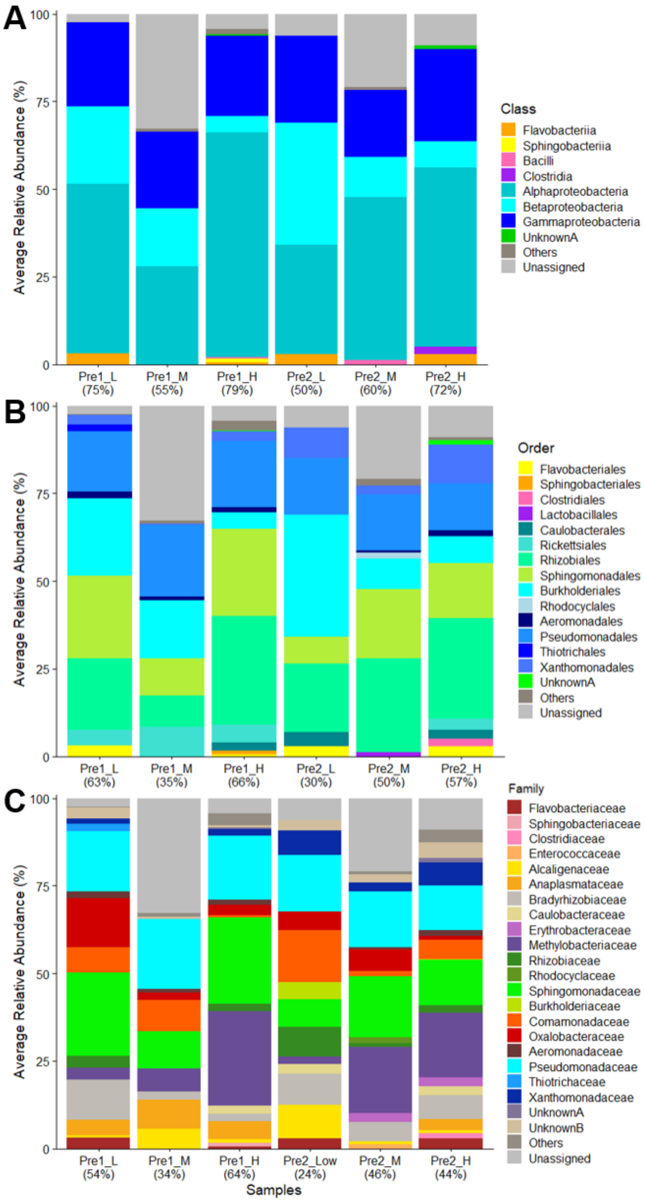


**Supplementary Figure 6 Bacterial A) Classes, B) Orders and C) Families in pre-flush biofilms of the Low-, Medium- and High-chlorine regimes.** Pre1 = Pre-Flush1, Pre-2=Pre-Flush2, L=Low-Chlorine, M=Medium-Chlorine, H=High-Chlorine. No difference in presence/absence (global-R≤0.089, p≥0.056) at any level. Percentage similarity between replicates of each sample point (n=5) shown in brackets. “Unassigned”=taxonomic information unavailable, UnknownA =Uncultured Bacteria, Unknown B = *Proteobacteria*, family unknown; “Others” incorporates taxa ≤1% total relative abundance, see Table S5.

N.b. Betaproteobacteria have been reclassified as the order *Betaproteobacteriales* within *Gammaproteobacteria* (Parks et al., 2018), however, previous nomenclature was maintained for clarity and consistency with previous studies.

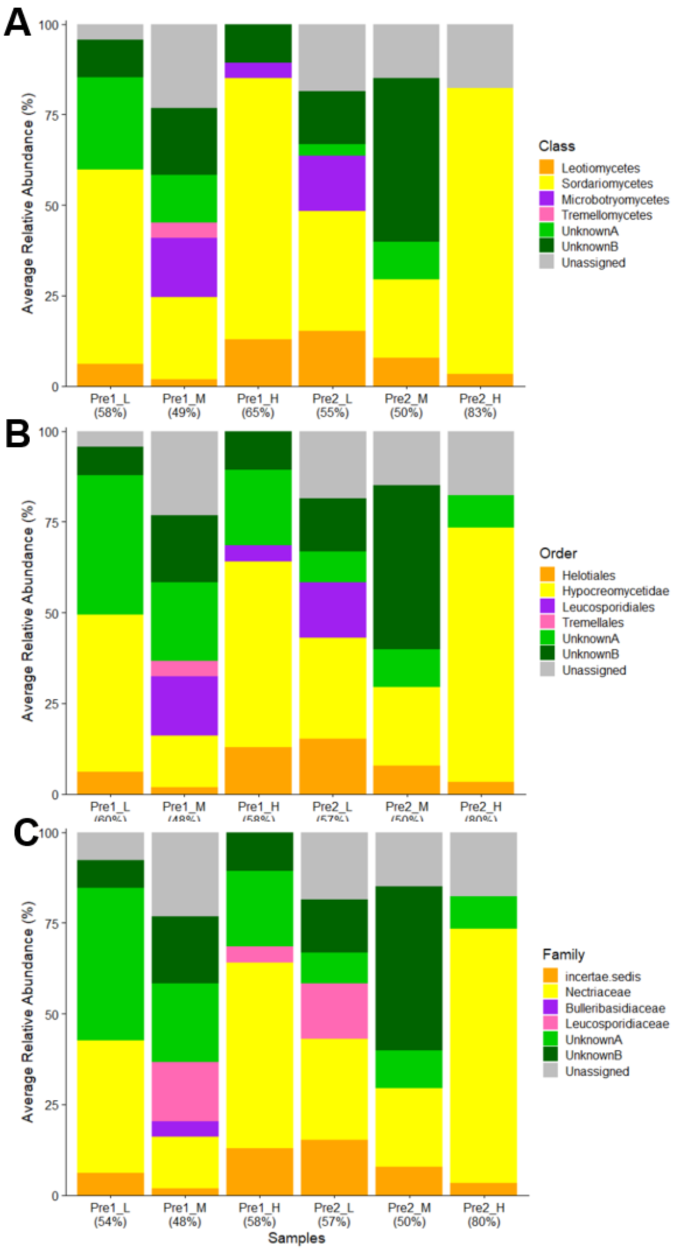


**Supplementary Figure 7 Relative abundance of fungal A) Classes, B) Orders and C) Families in pre-flush biofilms of the Low-, Medium- and High-chlorine regimes.** Pre1 = Pre-Flush1, Pre-2=Pre-Flush2, L=Low-Chlorine, M=Medium-Chlorine, H=High-Chlorine. For each plot n=5, percentage similarity between replicates of each sample point (n=5) shown in brackets. "Unassigned" refers to sequences for which taxonomic information was unavailable, UnknownA =assigned to Sub-kingdom *Dikarya*, UnknownB = Assigned to Fungi, no further information.

**Supplementary Table 1 Water quality parameters measured via spot samples during the Growth- and Regrowth-phases of the Low-, Medium- (Med) and High-chlorine regimes.** Means (and standard deviations) are shown.

| Instrument/ Analysis Method | Water Quality Parameter | Growth | | | Regrowth | | | UK Standard [C] |
|---|---|---|---|---|---|---|---|---|
| | | Low | Med | High | Low | Med | High | |
| ChloroSense Meter (Palintest, U.K.) | Free Chlorine (mg $l^{-1}$) [A] | 0.06 (0.07) | 0.45 (0.05) | 0.76 (0.17) | 0.03 (0.02) | 0.36 (0.05) | 0.72 (0.05) | None |
| | Total Chlorine (mg $l^{-1}$) [A] | 0.11 (0.08) | 0.55 (0.07) | 0.88 (0.17) | 0.06 (0.01) | 0.43 (0.06) | 0.81 (0.05) | Max 5.00 |
| | Temperature (°C) [A] | 12.7 (0.9) | 12.2 (1.0) | 12.7 (0.7) | 13.9 (0.3) | 13.7 (0.4) | 13.7 (0.3) | None |
| Inductively Coupled Plasma Optical Emission Spectroscopy (analysed at AlControl Laboratories, UK) | Iron (µg $l^{-1}$) [B] | 18.9 (3.9) | 21.5 (9.7) | 23.3 (7.7) | 20.6 (11.9) | 14.4 (4.6) | 15.4 (1.7) | 200.00 |
| | Manganese (µg $l^{-1}$) [B] | <3.6 (0.0) | 4.1 (1.0) | <3.6 (0.0) | <3.6 (0.0) | <3.6 (0.0) | <3.6 (0.0) | 50.00 |
| | Phosphorus (µg $l^{-1}$) [B] | 1274 (29) | 1273 (36) | 1263 (31) | 1320 (54) | 1313 (59) | 1317 (53) | |
| Hanna HI991003 portable multi-probe | pH [A] | 7.60 (0.34) | 8.10 (0.28) | 7.81 (0.51) | 7.8 (0.1) | 8.1 (0.2) | 7.9 (0.3) | 6.50 – 9.50 |
| | Oxidising Redox Potential (mV) [A] | 526 (34) | 612 (21) | 589 (53) | 515 (85) | 587 (30) | 597 (26) | None |
| Compact Turbimeter (Palintest, U.K.) | Turbidity (NTU) [A] | 0.05 (0.07) | 0.12 (0.19) | 0.04 (0.05) | 0.08 (0.21) | 0.11 (0.25) | 0.02 (0.03) | 1.00 – 4.00[D] |
| Microbiological content via flow cytometry, using a C6 flow cytometer (BD Accuri, U.K.) | Total Cell Concentration (cell $ml^{-1}$) [B] | 50913 (61877) | 1921 (1765) | 6400 (7237) | 125913 (54387) | 17050 (20407) | 7157 (4811) | None |
| | Intact Cell Concentration (cell $ml^{-1}$) [B] | 30948 (48635) | 1184 (1357) | 997 (1020) | 87670 (31216) | 3827 (1500) | 3340 (1613) | None |

[A] n=39, triplicate samples taken three times each week; [B] n=15, triplicate samples taken weekly; [C] U.K. standards based on Drinking Water Inspectorate (DWI) or European Union (EU) legislation, except for chlorine which is the World Health Organisation (WHO) standard as DWI and EU do not provide one; [D] Maximum values, water leaving a treatment plant must be ≤ 1 NTU, end point water ≤ 4 NTU.

**Supplementary Table 2 Results of statistical correlation tests applied to pre-flush biofilms.** (Data were not normally distributed, Spearman's Rank Correlation Co-efficient test was run in Rv3.5, n=9 for each test).

| **Sample Point** | **Correlation Test Parameters** | **Spearman's Rank Results** | | |
|---|---|---|---|---|
| | | **Rho** | **p-value** | **Slope** |
| Pre-Flush1 | Chlorine and TCC[A] | -0.896 | <0.001 | -440000 |
| | Chlorine and ICC[B] | -0.949 | <0.001 | -3000000 |
| | Chlorine and iron concentration | 1.000 | <0.001 | 0.33 |
| Pre-Flush2 | Chlorine and TCC[A] | -0.474 | 0.197 | -860000 |
| | Chlorine and ICC[B] | -0.474 | 0.197 | -490000 |
| | Chlorine and iron concentration | 1.000 | <0.001 | 1.3 |

[A]TCC=Total Cell Concentration; [B]Intact Cell Concentration

**Supplementary Table 3 Biofilm volumes and associated ratios of pre- and post-flush biofilms from Low-, Medium- and High-chlorine regimes.** Averages (median) presented (n=15) with the range (minimum-maximum).

| Sample Point | Chlorine regime | Biofilm Volume (1000 $\mu m^3$ per FOV)[A] | EPS:Cell [B] volume ratio (AU) [C] | Carbohydrate:Protein volume ratio (AU) [C] |
|---|---|---|---|---|
| Pre-Flush1 | Low | 1.46 (0.08 - 34.41) | 4.39 (0.02-36.36) | 0.01 (0.00-1.54) |
| | Medium | 6.50 (0.26 - 13.73) | 1.14 (0.28-37.76) | 0.01 (0.00-0.14) |
| | High | 2.08 (2.33 - 145.20) | 1.24 (0.08-31.33) | 0.42 (0.00-39.00) |
| Post-Flush1 | Low[D] | 6.11 (0.65 - 40.95) | 0.54 (0.06-28.47) | 1.01 (0.01-8.91) |
| | Medium | 3.72 (0.29 - 11.98) | 0.67 (0.03-32.11) | 5.12 (0.06-82.25) |
| | High | 1.99 (1.63 - 173.92) | 0.32 (0.00-13.12) | 0.31 (0.01-338.60) |
| Pre-Flush2 | Low | 21.35 (0.61 - 120.27) | 2.25 (0.06-11.30) | 0.52 (0.01-6.64) |
| | Medium | 2.85 (4.46 - 77.51) | 0.63[E] (0.06-107.21) | 0.82 (0.06-31.9) |
| | High | 14.96 (6.59 - 123.53) | 2.66 (0.09-28.55) | 1.98 (0.01-241.42) |
| Post-Flush2 | Low | 56.99 (1.57 - 83.76) | 1.30 (0.10-18.91) | 0.51 (0.02-2.90) |
| | Medium | 8.23 (0.37 - 83.76) | 5.50 (0.15-64.95) | 4.31 (0.11-61.02) |
| | High | 7.33 (0.08 - 34.41) | 3.86 (0.62-27.34) | 10.55 (0.19-243.50) |

FOV=field(s)-of-view, 420µm x 420µm; [A]Biofilm volume=carbohydrates+proteins+cells, before averaging, median and range of sums are presented; [B]EPS=carbohydrates+proteins; [C]First component divided by second, >1=greater volume of the first, <1=greater volume of the second; AU=arbitrary units. Ratios not calculated for FOV where a component was undetected, therefore n=7-15 for ratio data; [D]n=14; [E]Ratios more varied than other sample points (mean 8.82 AU).

**Supplementary Table 4 Ecological indices of the bacterial and fungal communities comprising biofilms from the Low, Medium and High chlorine regimes.** Means (and standard deviations) are shown (n=5, or n=4, see Methods 5.8).

| Taxa | Sample Point | Chlorine Regime[A] | Richness (Chao1) | Diversity (Shannon) | Evenness (Simpson-inverted) |
|---|---|---|---|---|---|
| Bacteria | Pre-Flush 1[C] | Low | 35 (24) [C] | 3.15 (1.00) [C] | 0.93 (0.07) [C] |
| | | Medium | 15 (14) [B] | 2.26 (1.00) [B] | 0.84 (0.13) [C] |
| | | High | 39 (25) [C] | 3.41 (0.61) [C] | 0.96 (0.02) [C] |
| | Post-Flush1 | Low | 29 (12) [C] | 3.21 (0.41) [C] | 0.95 (0.02) [C] |
| | | Medium | 77 (33) [B] | 4.17 (0.44)[B] | 0.98 (0.01) [C] |
| | | High | 73 (34) [C] | 4.10 (0.51) [C] | 0.98 (0.01) [C] |
| | Pre-Flush2 | Low | 14 (13) [C] | 1.91 (1.48) [C] | 0.66 (0.42) [C] |
| | | Medium | 28 (21) [C] | 2.88 (1.08) [C] | 0.90 (0.11) [C] |
| | | High | 29 (20) [C] | 3.09 (0.76) [C] | 0.94 (0.04) [C] |
| | Post-Flush2 | Low | 57 (39) [C] | 3.42 (1.57) [C] | 0.90 (0.16) [C] |
| | | Medium | 31 (15) [C] | 3.25 (0.47) [C] | 0.96 (0.02) [C] |
| | | High | 41 (17) [C] | 3.5 (0.96) [C] | 0.96 (0.03) [C] |
| Fungi | Pre-Flush 1 | Low | 4 (3) [C] | 1.13 (0.80) [C] | 0.57 (0.35) [C] |
| | | Medium | 7 (3) [C] | 1.77 (0.33) [C] | 0.81 (0.06) [C] |
| | | High | 5 (2) [C] | 1.46 (0.33) [C] | 0.74 (0.08) [C] |
| | Post-Flush1 | Low | 10 (6) [C] | 1.97 (0.74) [C] | 0.81 (0.15) [C] |
| | | Medium | 7 (3) [C] | 1.62 (0.68) [C] | 0.74 (0.21) [C] |
| | | High | 7 (4) [C] | 1.63 (0.55) [C] | 0.76 (0.12) [C] |
| | Pre-Flush2 | Low | 11 (4) [C] | 2.15 (0.41) [C] | 0.86 (0.06) [C] |
| | | Medium | 5 (2) [C] | 1.46 (0.36) [C] | 0.74 (0.09) [C] |
| | | High | 7 (2) [C] | 1.76 (0.32) [C] | 0.80 (0.06) [C] |
| | Post-Flush2 | Low | 8 (5) [C] | 1.75 (0.76) [C] | 0.76 (0.21) [C] |
| | | Medium | 9 (7) [C] | 1.83 (0.82) [C] | 0.78 (17) [C] |
| | | High | 9 (2) [C] | 2.03 (0.28) [C] | 0.85 (0.04) [C] |

[A] No significant differences in ecological indices were detected between chlorine regimes, at either time point, for bacteria ($F \geq 0.294$, $p \geq 0.111$) or fungi ($F \geq 0.026$, $p \geq 0.077$); [B] Difference ($t=-3.9$, $p=0.01$) but high variation; [C] No significant differences were detected between Pre- and Post-flush for Test 1 or Test 2 in any of the regimes (apart from indicated by B) for bacteria ($-2.47 \leq t \leq 0.48$, $0.060 \leq p \leq 0.901$) or fungi ($-1.75 \leq t \leq 1.07$, $0.126 \leq p \leq 0.809$).

**Supplementary Table 5 Taxonomic information for the category "Others" in Figure 5 and Supplementary Figure 6.**

| Graph (panel) | Taxonomic level | Constituents of "Others"<br>(≤1% total relative abundance of a sample) |
|---|---|---|
| Figure S6 (a) | Phyla_Class | *Actinobacteria_Actinobacteria*<br>*Bacteroidetes_Cytophagia*<br>*Firmicutes_Negativicutes* |
| Figure S6 (b) | Order | *Bacillales*<br>*Corynebacteriales*<br>*Cytophagales*<br>*Nitrosomonadales*<br>*Oceanospirillales*<br>*Propionibacteriales*<br>*Rhodobacterales*<br>*Selenomonadales* |
| Figure S6 (c) | Family | *Cytophagaceae*<br>*Halomonadaceae*<br>*Hyphomicrobiaceae*<br>*Moraxellaceae*<br>*Nocardiaceae*<br>*Nocardioidaceae*<br>*Paenibacillaceae*<br>*Peptococcaceae*<br>*Phyllobacteriaceae*<br>*Rhodobacteraceae*<br>Unknown B = (*Proteobacteria*; family unknown)<br>Unknown C = (*Firmicutes*; family unknown) |
| Figure 5 (c) | Genus | *Aeromicrobium*<br>*Afipia*<br>*Burkholderia*<br>*Candidatus.Odyssella*<br>*Comamonas*<br>*Desulfosporosinus*<br>*Halomonas*<br>*Herbaspirillum*<br>*Hyphomicrobium*<br>*Meganema*<br>*Methylophilus*<br>*Methylotenera*<br>*Paenibacillus*<br>*Pelomonas*<br>*Phenylobacterium*<br>*Phyllobacterium*<br>*Polynucleobacter*<br>*Rhodobacter*<br>*Rhodococcus*<br>*Simplicispira*<br>*Spirosoma*<br>*Tardiphaga*<br>*Xanthomonas*<br>*Zymomonas*<br>*Acidobacteria;* genus unknown<br>*Bacteroidetes;* genus unknown |

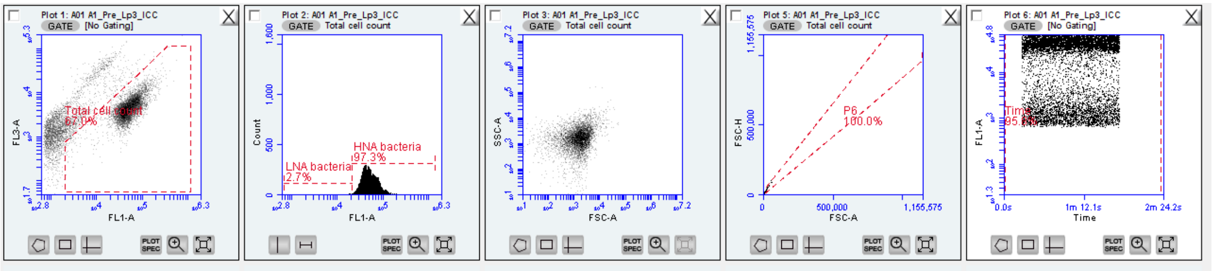


**Supplementary Figure 8 Plots exemplifying the gating strategy used for the flow cytometry analysis in this study, as referenced in the npj Reporting Summary.** Provided as an unadjusted screen shot from the BD software for the BD Accuri C6 flow cytometer. The last two plots were added to the standard template to provide additional information. The threshold was set on FSC-H and set at 800.
